# Multibreed genomic prediction using summary statistics and a breed-origin-of-alleles approach

**DOI:** 10.1038/s41437-023-00619-4

**Published:** 2023-05-25

**Authors:** J. B. Clasen, W. F. Fikse, G. Su, E. Karaman

**Affiliations:** 1 Department of Animal Breeding and Genetics, Swedish University of Agricultural Sciences, Box 7023, 75007 Uppsala, Sweden; 2 Center for Quantitative Genetics and Genomics, Aarhus University, C. F. Møllers Allé 8, DK-8000 Aarhus, Denmark; 3 Växa Sverige, Swedish University of Agricultural Sciences, Ulls väg 26, 756 51 Uppsala, Sweden

**Keywords:** Animal breeding, Agricultural genetics

## Abstract

Because of an increasing interest in crossbreeding between dairy breeds in dairy cattle herds, farmers are requesting breeding values for crossbred animals. However, genomically enhanced breeding values are difficult to predict in crossbred populations because the genetic make-up of crossbred individuals is unlikely to follow the same pattern as for purebreds. Furthermore, sharing genotype and phenotype information between breed populations are not always possible, which means that genetic merit (GM) for crossbred animals may be predicted without the information needed from some pure breeds, resulting in low prediction accuracy. This simulation study investigated the consequences of using summary statistics from single-breed genomic predictions for some or all pure breeds in two- and three-breed rotational crosses, rather than their raw data. A genomic prediction model taking into account the breed-origin of alleles (BOA) was considered. Because of a high genomic correlation between the breeds simulated (0.62–0.87), the prediction accuracies using the BOA approach were similar to a joint model, assuming homogeneous SNP effects for these breeds. Having a reference population with summary statistics available from all pure breeds and full phenotype and genotype information from crossbreds yielded almost as high prediction accuracies (0.720–0.768) as having a reference population with full information from all pure breeds and crossbreds (0.753–0.789). Lacking information from the pure breeds yielded much lower prediction accuracies (0.590–0.676). Furthermore, including crossbred animals in a combined reference population also benefitted prediction accuracies in the purebred animals, especially for the smallest breed population.

## Introduction

Crossbreeding is the central breeding strategy in commercial poultry and pig production, due to the benefits of heterosis. However, except for New Zealand, crossbreeding between dairy cattle breeds has been rarely utilized worldwide. Nevertheless, crossbreeding has shown positive effects, primarily on functional traits, such as fertility and health (Sørensen et al. 2008; Shonka-Martin et al. 2019), which can be economically beneficial in dairy herds (Clasen et al. 2020). As a result, the interest in crossbreeding in dairy herds is increasing, and farmers request genomically enhanced breeding values (GEBVs) for crossbred animals (Clasen et al. 2021).

Genomic evaluation has revolutionized dairy cattle breeding within the last decade and has become the primary way of selecting dairy cattle sires in commercial dairy breeding (Hutchison et al. 2014; Mäntysaari et al. 2020). With GEBVs for purebred dairy cattle, it is possible to select future breeding sires at a very young age, with prediction accuracies nearly as high as accuracies for daughter-proven AI bulls for any trait. Also, genomic testing of virgin heifers is a valuable tool for the farmers to select future replacement heifers more accurately (Hjortø et al. 2015; Calus et al. 2015). As the cost of a genomic test has reduced rapidly in the last few years, genomic selection of females in individual dairy herds is becoming increasingly attractive for farmers (Wiggans et al. 2017; Bengtsson et al. 2020). However, estimating GEBVs for crossbred animals is not as straightforward as for purebred animals.

Crossbred animals may have genomic breed proportions (GBP) different from breed proportions estimated from pedigrees, especially if they are crossbred through many generations, e.g., in a rotational crossbreeding system (Wu et al. 2020). Thus, the genetic makeup of crossbred animals may differ substantially even when pedigree-based breed proportions are the same, and crossbred animals may differ too much amongst each other to be treated as an individual breed as a whole.

There are different suggestions on how to estimate GMs for crossbred animals. Combining data from several breeds in a multibreed reference population often considers homogeneous SNP effects across all breeds (Hayes et al. 2009). For this approach to benefit (purebred) GMs, the genetic correlation between the breeds for the given trait needs to be high (Brøndum et al. 2011; Lund et al. 2011; Karoui et al. 2012). Including crossbred animals in the combined reference population for multibreed prediction has benefited GMs (Khansefid et al. 2020). However, different breeds and their crosses do not necessarily have the same effects of quantitative trait loci (QTL). Furthermore, even if the QTL effects are the same, the linkage disequilibrium (LD) between genome-wide single nucleotide polymorphisms (SNPs) markers and QTL may differ (Hayes et al. 2009; Vandenplas et al. 2016). Therefore the multibreed prediction model without accounting for the difference in SNP effects between breeds may not be accurate, especially if the breeds are relatively distant (Brøndum et al. 2011; Erbe et al. 2012).

Another approach to genomic evaluation of crossbred is to estimate SNP effects for individual pure breeds and then weigh them accordingly with the crossbred GBP. This method is described by VanRaden et al. (2020) and is the basis for crossbred GMs implemented in the US dairy cattle breeding evaluation since April 2019 (Wiggans et al. 2019). VanRaden et al. (2020) evaluated the method, and estimated accuracies of genomic predicted transmitting ability up to 0.52 for milk yield. However, a limitation of this method is that it cannot exploit crossbred animals in the reference population. Furthermore, the methods assume the same GBP for each SNP, which does not reflect the diverse genomic profile of crossbreds (Ibánẽz-Escriche et al. 2009; Sevillano et al. 2017).

Tracing each allele back to its breed origin (BOA) and using the estimated SNP effect for that breed yields a potentially higher prediction accuracy for crossbred animals than assuming homogeneous SNP effects (Vandenplas et al. 2016; Sevillano et al. 2018). In a simulation study, Karaman et al. (2021) proposed a multibreed model that can accommodate a reference population of both purebred and crossbred animals considering BOA for simultaneous evaluation of purebred and crossbred selection candidates. The prediction accuracies for three-breed crosses in a rotational crossbreeding system were similar to or higher than the traditional multibreed predictions, which combined data from all animals and assumed homogeneous SNP effects across all breeds. The benefit of their approach depended on the underlying QTL correlation between the main breeds.

Another issue for estimating GMs in crossbred animals is the limited availability of genotype and phenotype data on purebreds if foreign sire breeds are used in the crossbreeding system. A real example is a three-breed rotational system, crossing Holstein, Swedish Red, and French Montbéliarde (ProCross; www.procross.info). The first two breeds have populations in the Nordic countries (Denmark, Finland, and Sweden) and Montbéliarde is primarily found in France. The Nordic countries and France may have the advantage of having breed populations of two breeds, while any other country may only have a population of Holstein cattle. Sharing information between countries may be the most optimal (Lund et al. 2011; Jorjani et al. 2012), but political barriers and different handling of SNP information make it challenging (Tenopir et al. 2011; Liu and Goddard 2018). Hence, the prediction of GMs for crossbred animals may need to be done without SNP and phenotypic data from one or several purebreds in the crossbreeding strategy.

In human genetics, large amounts of data from a single population are rarely available for genomic predictions of, e.g., inheritable diseases. Instead, summary statistics obtained from separate populations are joined into a meta-analysis, which makes it possible to utilize information of multiple populations without the necessity of sharing individual data (e.g., Maier et al. 2018; Lloyd-Jones et al. 2019). In livestock, genomic prediction using summary statistics including estimated allele substitution effects of markers and the prediction error variances (PEV) has shown promising results (Vandenplas et al. 2018) in a combined analysis of multiple breeds. In dairy cattle, such an approach is being under development in the Interbull SNPMace project (Jighly et al. 2019).

In this simulation study, we performed genomic prediction using a breed-of-origin model proposed by Karaman et al. (2021) and investigated the consequences of using summary statistics in contrast to having full data or lacking information from some of the breeds in two- and three-breed rotational crossbreeding systems.

## Materials and methods

### Data simulation

**Fig. 1:**
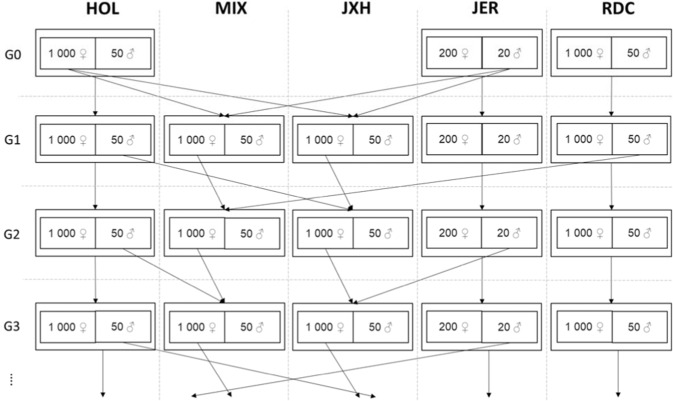
Simulation of mating in three pure breed populations. Danish Holstein (HOL), Danish Jersey (JER), and Swedish Red (RDC), a two-breed rotational crossbred population (JXH), and a three-breed rotational crossbred population (MIX). For each generation, 1000 females of each breed (200 in JER) and crossbred are randomly mated to 50 males (20 in JER). Crossbred populations uses males from the pure breed populations, and crossbred males are not used for breeding.

The simulation of data was described in Karaman et al. (2021) and data were generated using the same base population individuals. Briefly, genotype data were available at 51,477 loci for three populations, of which we created a subset of 1050 Danish Holstein (HOL), 1050 Swedish Red (RDC), and 220 Danish Jersey (JER). However, we only considered SNPs on the first five chromosomes (12,664 SNPs) to reduce the computational demand. Twelve generations of purebreds were created by randomly mating 1000 (HOL and RDC) and 200 (JER) females with 50 and 20 males in each generation, respectively (Fig. 1). The 50 HOL and RDC sires were mated each with 20 females, while the 20 JER sires were mated each with 10 females. The dams were randomly assigned to the sires. Each mating resulted in one offspring, except for one mating per sire that yielded two offspring, to keep the population sizes constant across the generations. Hence, each generation consisted of 1050 (HOL and RDC) and 220 (JER) animals. In each generation, 1000 of all HOL and RDC offspring were randomly identified as female and 50 as males. Correspondingly, for JER, 200 were identified as female and 20 as males.

Two different crossbred populations were created based on the purebred animals initially generated (Fig. 1). A three-breed rotational crossbreeding system was used to create an admixed HOL × JER × RDC population (MIX), starting with mating 1000 HOL females with 20 JER males from the base generation (generation 0). At the next generation, 1000 MIX females (all with a JER sire) were crossed with respectively 50 RDC males. In the last stage of the rational system, 1000 MIX females (all with a RDC sire) were mated with 50 HOL males. This cycle continued and resulted in 1050 animals in each generation in the MIX population. Similarly, a two-breed rotational crossbreeding system was used to create JER × HOL crossbreds (JXH), alternating between 20 JER and 50 HOL sires. The JXH population also consisted of 1050 animals per generation. Consequently, all MIX and JXH animals in the 12th generation were sired by HOL. A similar strategy as in the pure breeds was applied to keep the population sizes constant across generations of MIX and JXH. When MIX and JXH females were mated to HOL males, each HOL male was mated to 20 females of each MIX and JXH. The same applied to RDC males in the MIX population. When MIX and JXH females were mated to JER males, each JER males were mated to 50 females to keep the population size.

All animals had known breed of origin of alleles traced back to the base population at generation 0. To generate phenotypes, 500 QTL were selected randomly among the SNPs with minor allele frequencies (MAF) across the breeds between 0.01 and 0.30, as described by Karaman et al. (2021). The genetic value (*g*_i_) of individual *i* were simulated following a similar strategy as Akdemir et al. (2017) as follows. First, a principal component (PC) analysis was performed using the genotypes of all animals at the 500 QTL, and the first two PCs were normalized, and used as indicators of the genetic background, with which all QTL effects interacted. Three coefficients, *a*_*k*_, *b*_*k*_ and *c*_*k*_, were introduced to account for genetic background together with PCA. The *a*_*k*_ accounted for differences among the breeds on PC1, and *b*_*k*_ and *c*_*k*_ on PC2. Second, genotypic values were simulated using:

if PC1 < 0

$${g}_{i}=\mathop{\sum }\limits_{k=1}^{500}({x}_{ik}\ast {a}_{k}+{x}_{ik}\ast PC{2}_{i}\ast {b}_{k})$$

else

$${g}_{i}=\mathop{\sum }\limits_{k=1}^{500}({x}_{ik}\ast {a}_{k}+{x}_{ik}\ast PC{2}_{i}\ast {c}_{k})$$

where *x*_*ik*_ is the genotype at locus *k*, ${a}_{k} \sim N(0,1)$, *b*_*k*_ ~ *N*(0, 0.2) and *c*_*k*_ ~ *N*(0, 0.2) (values of the parameters for N(.) were inferred from Duenk et al. 2020). Thus, with this setting, each individual had a unique set of QTL effects, *α*_*ik*_ = *a*_*k*_ + ${PC2}_{i}\, *\,{b}_{k}$ or *α*_*ik*_ = *a*_*k*_ + ${PC2}_{i}\, *\, {C}_{k}$

The mean value of *α*_*ik*_ was used to compute breed-specific QTL substitution effects at each locus, *α*_*k*_, which were used to compute additive genetic values for the base population individuals. The *a*_*k*_, *b*_*k*_, *c*_*k*_, and accordingly, *α*_*k*_ were scaled such that the mean of the within-breed variances of the additive genetic values was 100.

All animals had an environmental effect, sampled from a normal distribution These environmental effects are then added to their genotypic value to form the phenotype, hence both males and females had phenotypes generated. The broad sense heritability of the simulated trait varied slightly among the breeds but was around 0.42, averaged over the base populations. In total 25 replicates were generated.

The simulated genetic correlations (see Karaman et al. (2021) for computations) between the breeds were 0.87 between HOL and RDC, 0.62 between HOL and JER, and 0.68 between RDC and JER, for the base populations (generation 0).

Employing different reference populations and statistical models, we investigated several scenarios for genomic prediction of five test populations.

### Reference and test populations

Animals from generations 9, 10, and 11 from each pure breed and crossbred population were used to form the reference populations. Thus, 660 JER animals and 3150 animals from each of the four other populations were utilized. Putting together animals from all five populations, the combined reference population consisted of 13,260 animals, but in other scenarios animals from only some of the five populations were considered in the reference population, as detailed below.

The animals used in the test population were all from generation 12, i.e., 220 JER and 1050 animals of each of the four other populations. The GBP of crossbred animals were computed from alleles traced back to the breed of origin (Table 1).

**Table 1:** Average genomic breed proportions of Danish Holstein (HOL), Swedish Red (RDC), and Danish Jersey (JER) in test populations (generation 12) of three-breed rotational crosses (MIX) and Jersey × Holstein rotational crosses (JXH), and in MIX, JXH, and combined^a^ reference populations (generations 9, 10 and 11) with standard deviations in subscript.

	HOL	RDC	JER
Reference population			
MIX	0.335_0.189_	0.333_0.188_	0.331_0.190_
JXH	0.442_0.171_	–	0.558_0.171_
Combined	0.422_0.415_	0.316_0.385_	0.261_0.312_
Test population			
MIX	0.573_0.046_	0.282_0.074_	0.145_0.063_
JXH	0.667_0.069_	–	0.333_0.069_

^a^Combined reference population including 3150 of each HOL, RDC, MIX, JXH, and 660 JER.

### Statistical models for estimation of SNP effects

#### Homogeneous SNP effect model

We used the following statistical model to estimate SNP effects, separately within each population:

1 $${{\bf{y}}}_{{\bf{i}}}={\bf{1}}{{{\upmu }}}_{{\bf{i}}}+{{\bf{M}}}_{{\bf{i}}}{{\boldsymbol{\beta }}}_{{\bf{i}}}+{{\bf{e}}}_{{\bf{i}}}$$

where *i* stands for the breeds HOL, JER, and RDC. When data from multiple populations (*J*) were analyzed jointly, the combined reference population was treated as a single homogeneous population using the following model:

2 $${{\bf{y}}}_{{\rm{J}}}=1{{\rm{\mu }}}_{{\rm{J}}}+{\bf{X}}{{\bf{b}}}_{{\rm{J}}}+{\bf{M}}{{\boldsymbol{\beta }}}_{{\rm{J}}}+{{\bf{e}}}_{{\rm{J}}}$$

In models (1) and (2), **y** is the vectors of phenotypes, **1** is a vector of 1 s, *µ* is the overall means, **X** is the matrix of (centered) estimated genomic breed proportions computed from genomic data, **b**_J_ is the vector of fixed breed effects, **M** is the matrices of centered genotypes where centering was based on the column means, **β**_J_ is the vectors of SNP effects, and **e** is the vectors of residuals.

#### Heterogeneous SNP effect model

For the BOA approach, model (2) was extended with a genetic component for each breed instead of one joint (Karaman et al. 2021), as follows:

3 $${\bf{y}}=1{{\rm{\mu }}}_{{\rm{BOA}}}+{\bf{X}}{{\bf{b}}}_{{\rm{BOA}}}+{{\bf{M}}}_{1}{{\boldsymbol{\beta }}}_{{\rm{BOA}},{\rm{HOL}}}+{{\bf{M}}}_{2}{{\boldsymbol{\beta }}}_{{\rm{BOA}},{\rm{JER}}}+{{\bf{M}}}_{3}{{\boldsymbol{\beta }}}_{{\rm{BOA}},{\rm{RDC}}}+{{\bf{e}}}_{{\rm{BOA}}}$$

where **y**, **1**, *μ*_*BOA*_, and **X** are as described in models (1) and (2), **M**_1_, **M**_2_, and **M**_3_ are the matrices of breed-specific allele content of SNPs for HOL, JER, and RDC, respectively. For example, the value at a locus in **M**_1_ is the number of counted (0, 1, or 2) reference alleles originating from HOL, and the same logic was applied to **M**_2_ and **M**_3_. The matrices were column-centered before the analysis. The **β**s are the vectors of SNP effects for HOL, JER, and RDC, respectively, and **e** is the vector of residuals.

We used a Bayesian approach for estimating the dispersion and location parameters, which required assigning prior distributions to the unknowns of the model. For models (1) and (2) a normal distribution with null mean and a common variance was assigned for all SNPs, ${{\boldsymbol{\beta }}}_{i}|{{\rm{\sigma }}}_{{{\rm{\beta }}}_{i}}^{2} \sim {\rm{N}}(0,\,{\bf{I}}{{\rm{\sigma }}}_{{{\rm{\beta }}}_{i}}^{2})$ or ${{\boldsymbol{\beta }}}_{J}|{{\rm{\sigma }}}_{{{\rm{\beta }}}_{J}}^{2} \sim {\rm{N}}({\bf{0}},\,{\bf{I}}{{\rm{\sigma }}}_{{{\rm{\beta }}}_{J}}^{2})$. For model (3), a normal distribution prior was assigned to the vectors of SNP effects for each breed: ${{\boldsymbol{\beta }}}_{{\rm{BOA}},{\rm{i}}}|{{\rm{\sigma }}}_{{{\rm{\beta }}}_{{\rm{BOA}},{\rm{i}}}}^{2} \sim {\rm{N}}(0,{\bf{I}}{{\rm{\sigma }}}_{{{\rm{\beta }}}_{{\rm{BOA}},{\rm{i}}}}^{2})$, where *i* stands for the breeds HOL, JER, and RDC. Residuals were assumed to follow a normal prior distribution, *N*(**0**, **R**) where **R** was a diagonal matrix of residual variances, in all models. For all models, SNP and residual variances were assumed to follow a scaled inverted chi-square prior with degrees of freedom (df) and scale (S) parameters. For the SNP variances, priors were ${{\rm{\sigma }}}_{{{\rm{\beta }}}_{i}}^{2}|{\rm{df}},{{\rm{S}}}_{i} \sim {{\rm{\chi }}}^{-2}({\rm{df}},{{\rm{S}}}_{i})$,

$${\sigma }_{\beta {\rm{j}}}^{2}|{{\rm{df}}}_{{\rm{J}}},\,{{\rm{S}}}_{\beta {\rm{J}}}\sim{\chi }^{-2}({\rm{df}},\,{{\rm{S}}}_{\beta {\rm{J}}})\,{\rm{or}}\,{\sigma }_{\beta {\rm{BOA}},{\rm{i}}}^{2}|{\rm{df}},\,{{\rm{S}}}_{\beta {\rm{BOA}},{\rm{i}}} \sim {\chi }^{-2}({\rm{df}},{{\rm{S}}}_{\beta {\rm{BOA}},{\rm{i}}})$$

for models (1), (2), or (3), respectively. For the residual variances, priors for model (1) was ${{\rm{\sigma }}}_{{{\rm{e}}}_{i}}^{2}|{\rm{df}},\,{{\rm{S}}}_{{{\rm{e}}}_{i}} \sim {{\rm{\chi }}}^{-2}({\rm{df}},{{\rm{S}}}_{{{\rm{e}}}_{i}})$, and the priors for models 2 and 3 were, ${{\rm{\sigma }}}_{{{\rm{e}}}_{{\rm{J}}}}^{2}|{\rm{df}},{{\rm{S}}}_{{{\rm{e}}}_{{\rm{J}}}} \sim {{\rm{\chi }}}^{-2}({\rm{df}},{{\rm{S}}}_{{{\rm{e}}}_{{\rm{J}}}})$, and ${{\rm{\sigma }}}_{{{\rm{e}}}_{{\rm{BOA}}}}^{2}|{\rm{df}},{{\rm{S}}}_{{{\rm{e}}}_{{\rm{BOA}}}} \sim {{\rm{\chi }}}^{-2}({\rm{df}},{{\rm{S}}}_{{{\rm{e}}}_{{\rm{BOA}}}})$, respectively. The value of degree of freedom parameter (df) was set to 4 in all cases. The values of scale parameters (S) were derived from the expected value of a scaled inverted chi-square distributed random variable using ${\rm{S}}=\frac{{{\rm{\sigma }}}^{2}({\rm{df}}-2)}{{\rm{df}}}$ (Habier et al. 2011) where the σ^2^ can be an estimate of the variance of SNP effects or residuals (Karaman et al. 2021). The fixed effects were assumed to follow a flat prior distribution.

#### Summary statistics

For the analyses using summary statistics, the prior distributions assigned to the SNP effects in models (2) and (3) were reformulated with summary statistics obtained from separate analysis of individual pure breeds (model (1), see Supplementary file 1). To this end, we only needed prediction error (co)variances (PEC) of SNP effects, number of animals, and their mean phenotypes, from the individual pure breed analysis. We assumed that PECs are diagonal matrices whose diagonal elements were the posterior variances of the SNP effects from the individual pure breed analysis. Crossbred animals had always phenotypes and genotypes, whereas the availability of summary statistics or actual data for the pure breeds varied as described earlier.

When only the data from crossbred animals (a) are available, model (2) can be written as

4 $${{\bf{y}}}_{{\rm{a}}}=1{{\rm{\mu }}}_{{\rm{J}}}+{{\bf{X}}}_{{\rm{a}}}{{\bf{b}}}_{{\bf{J}}}+{\bf{M}}{\boldsymbol{\beta }}+{{\bf{e}}}_{{\rm{a}}}$$

Then the information from summary statistics for the pure breeds can be used to form normal (N) and scaled inverted chi-square $\left({{{\upchi}}}^{-2}\right)$ prior distributions as follows:

$$\begin{array}{ll}{{{\upmu }}}_{{\rm{J}}} \sim {\rm{N}}\left\{\frac{1}{{{\rm{n}}}_{{\rm{HOL}}}+{{\rm{n}}}_{{\rm{JER}}}+{{\rm{n}}}_{{\rm{RDC}}}}\left(\right.{{\rm{n}}}_{{\rm{HOL}}}{\overline{{\rm{y}}}}_{{\rm{HOL}}}+{{\rm{n}}}_{{\rm{JER}}}{\overline{{\rm{y}}}}_{{\rm{JER}}}\right.\\ \qquad\qquad \left.+{{\rm{n}}}_{{\rm{RDC}}}{\overline{{\rm{y}}}}_{{\rm{RDC}}}\left)\right., \frac{1}{{{\rm{n}}}_{{\rm{HOL}}}+{{\rm{n}}}_{{\rm{JER}}}+{{\rm{n}}}_{{\rm{RDC}}}}{{{\sigma }}}_{{{\rm{e}}}_{{\rm{J}}}}^{2}\right\}\end{array}$$

$${{\bf{b}}}_{{\rm{J}}} \sim {\rm{N}}\left\{\begin{array}{c}{\left[\begin{array}{ccc}{{\rm{n}}}_{{\rm{HOL}}} & 0 & 0\\ 0 & {{\rm{n}}}_{{\rm{JER}}} & 0\\ 0 & 0 & {{\rm{n}}}_{{\rm{RDC}}}\end{array}\right]}^{-1}\left[\begin{array}{c}{{\rm{n}}}_{{\rm{HOL}}}{\bar{{\rm{y}}}}_{{\rm{HOL}}}\\ {{\rm{n}}}_{{\rm{JER}}}{\bar{{\rm{y}}}}_{{\rm{JER}}}\\ {{\rm{n}}}_{{\rm{RDC}}}{\bar{{\rm{y}}}}_{{\rm{RDC}}}\end{array}\right], {\left[\begin{array}{ccc}{{\rm{n}}}_{{\rm{HOL}}} & 0 & 0\\ 0 & {{\rm{n}}}_{{\rm{JER}}} & 0\\ 0 & 0 & {{\rm{n}}}_{{\rm{RDC}}}\end{array}\right]}^{-1}{{\rm{\sigma }}}_{{{\rm{e}}}_{{\rm{J}}}}^{2}\end{array}\right\}$$

$${{{\upbeta }}}_{{\rm{J}}} \sim {\rm{N}}\left\{\begin{array}{c}{\left[\displaystyle\sum {\rm{PEC}}{({\tilde{{{\upbeta }}}}_{{\rm{i}}})}^{-1}-\displaystyle\sum {{\bf{B}}}_{{\rm{i}}}^{-1}\right]}^{-1}\left[\displaystyle\sum {\rm{PEC}}{({\tilde{{{\upbeta }}}}_{{\rm{i}}})}^{-1}{\tilde{{{\upbeta }}}}_{{\rm{i}}}\right], {\left[\displaystyle\sum {\rm{PEC}}{({\tilde{{{\upbeta }}}}_{{\rm{i}}})}^{-1}-\displaystyle\sum {{\bf{B}}}_{{\rm{i}}}^{-1}\right]}^{-1}\end{array}\right\}$$

$${{{\upbeta }}}_{{\rm{J}}} \sim {\rm{N}}\left({\bf{0}},{\bf{I}}{{{\upsigma }}}_{{{{\upbeta }}}_{{\rm{J}}}}^{2}\right)$$

$${{\bf{e}}}_{{\rm{a}}} \sim {\rm{N}}\left({\bf{0}},{{\bf{D}}}_{{\rm{a}}}{{{\upsigma }}}_{{{\rm{e}}}_{J}}^{2}\right)$$

$${{{\upsigma }}}_{{{{\upbeta }}}_{{\rm{J}}}}^{2} \sim {{{\upchi }}}^{-2}\left({\rm{df}},{{\rm{S}}}_{{{{\upbeta }}}_{{\rm{J}}}}\right)$$

$${{{\upsigma }}}_{{\rm{e}}}^{2} \sim {{{\upchi }}}^{-2}\left({\rm{df}},{{\rm{S}}}_{{{\rm{e}}}_{{\rm{J}}}}\right)$$

When only the data from crossbred animals are available, model (3) can be written as

5 $${{\bf{y}}}_{{\rm{a}}}=1{{\rm{\mu }}}_{{\rm{BOA}}}+{{\bf{X}}}_{{\rm{a}}}{{\bf{b}}}_{{\rm{BOA}}}+[\begin{array}{ccc}{{\rm{M}}}_{{\rm{a}},1} & {{\rm{M}}}_{{\rm{a}},2} & {{\rm{M}}}_{{\rm{a}},3}\end{array}]\left[\begin{array}{c}{{\rm{\beta }}}_{{\rm{BOA}},{\rm{HOL}}}\\ {{\rm{\beta }}}_{{\rm{BOA}},{\rm{JER}}}\\ {{\rm{\beta }}}_{{\rm{BOA}},{\rm{RDC}}}\end{array}\right]+{{\bf{e}}}_{{\rm{a}}}$$

Then the information from summary statistics for the pure breeds can be used to form prior distributions as follows:

$$\begin{array}{ll}{{{\upmu }}}_{{\rm{BOA}}} \sim {\rm{N}}\left\{\frac{1}{{{\rm{n}}}_{{\rm{HOL}}}+{{\rm{n}}}_{{\rm{JER}}}+{{\rm{n}}}_{{\rm{RDC}}}}\left(\right.{{\rm{n}}}_{{\rm{HOL}}}{\bar{{\rm{y}}}}_{{\rm{HOL}}}+{{\rm{n}}}_{{\rm{JER}}}{\bar{{\rm{y}}}}_{{\rm{JER}}}\right.\\ \qquad\qquad \left.+\,{{\rm{n}}}_{{\rm{RDC}}}{\bar{{\rm{y}}}}_{{\rm{RDC}}}\left)\right., \frac{1}{{{\rm{n}}}_{{\rm{HOL}}}+{{\rm{n}}}_{{\rm{JER}}}+{{\rm{n}}}_{{\rm{RDC}}}}{{\rm{\sigma }}}_{{\rm{e}}}^{2}\right\}\end{array}$$

$${{\bf{b}}}_{{\rm{BOA}}} \sim {\rm{N}}\left\{\begin{array}{c}{\left[\begin{array}{ccc}{{\rm{n}}}_{{\rm{HOL}}} & 0 & 0\\ 0 & {{\rm{n}}}_{{\rm{JER}}} & 0\\ 0 & 0 & {{\rm{n}}}_{{\rm{RDC}}}\end{array}\right]}^{-1}\left[\begin{array}{c}{{\rm{n}}}_{{\rm{HOL}}}{\bar{{\rm{y}}}}_{{\rm{HOL}}}\\ {{\rm{n}}}_{{\rm{JER}}}{\bar{{\rm{y}}}}_{{\rm{JER}}}\\ {{\rm{n}}}_{{\rm{RDC}}}{\bar{{\rm{y}}}}_{{\rm{RDC}}}\end{array}\right], {\left[\begin{array}{ccc}{{\rm{n}}}_{{\rm{HOL}}} & 0 & 0\\ 0 & {{\rm{n}}}_{{\rm{JER}}} & 0\\ 0 & 0 & {{\rm{n}}}_{{\rm{RDC}}}\end{array}\right]}^{-1}{{\rm{\sigma }}}_{{\rm{e}}}^{2}\end{array}\right\}$$

$${{{\upbeta }}}_{{\rm{BOA}},{\rm{i}}} \sim {\rm{N}}\left\{\begin{array}{c}{\left[{\rm{PEC}}{({\tilde{{{\upbeta }}}}_{{\rm{i}}})}^{-1}-{{\bf{B}}}_{{\rm{i}}}^{-1}\right]}^{-1}\left[{\rm{PEC}}{({\tilde{{{\beta }}}}_{{\rm{i}}})}^{-1}{\tilde{{{\upbeta }}}}_{{\rm{i}}}\right], {\left[{\rm{PEC}}{({\tilde{{{\upbeta }}}}_{{\rm{i}}})}^{-1}-{{\bf{B}}}_{{\rm{i}}}^{-1}\right]}^{-1}\end{array}\right\}$$

$${{{\upbeta }}}_{{\rm{BOA}},{\rm{i}}} \sim {\rm{N}}\left({\bf{0}},{\bf{I}}{{{\upsigma }}}_{{{{\upbeta }}}_{{\rm{BOA}},{\rm{i}}}}^{2}\right)$$

$${{\bf{e}}}_{{\rm{a}}} \sim {\rm{N}}\left({\bf{0}},{{\bf{D}}}_{{\rm{a}}}{{{\upsigma }}}_{{\rm{e}}}^{2}\right)$$

$${{{\upsigma }}}_{{{{\upbeta }}}_{{\rm{BOA}},{\rm{i}}}}^{2} \sim {{{\upchi }}}^{-2}\left({\rm{df}},{{\rm{S}}}_{{{{\upbeta }}}_{{\rm{BOA}},{\rm{i}}}}\right)$$

$${{{\upsigma }}}_{{\rm{e}}}^{2} \sim {{{\upchi }}}^{-2}({{\rm{v}}}_{{\rm{e}}},{{\rm{S}}}_{{\rm{e}}})$$

In the above equations, ${\tilde{{\boldsymbol{\beta }}}}_{{\rm{i}}}$ is the vector of previously estimated SNP effects, and PEC(${\tilde{{\boldsymbol{\beta }}}}_{{\rm{i}}}$) is a diagonal matrix whose diagonal elements were the variances of the posterior samples of SNP effects, as explained above, from the separate analysis of breed *i*. The ${{\bf{B}}}_{{\rm{i}}}^{-1}$ is a diagonal matrix with diagonal elements being the mean of the posterior distribution of SNP variances, also from the separate analysis of breed *i*. The parameters for the above prior distributions defined for the analysis using summary statistics were set in the same manner as in the previously described corresponding full data analysis.

### Prediction of GM

Genetic merit for each test population were estimated using the SNP solutions from the separate analysis of individual purebred and crossbred reference populations (referred to as “within-breed approach”). This was referred to as within-breed approach because the reference and test populations are either from the same breed (e.g., predicting HOL GM with SNP solutions for HOL) or each belonging to a single population (e.g., predicting HOL GM with SNP solutions for JER).

The MIX and JXH test populations had also GMs estimated using the BOA approach based on solutions from each purebred reference population (HOL, RDC, and JER) and by tracing each SNP allele of the crossbred test animal back to its (pure) breed of origin (HOL, RDC, or JER) (referred to as “pure-BOA approach”). Hence, in the analysis using the pure-BOA approach, MIX, and JXH were not included in the reference population.

GMs were also predicted with two approaches using SNP estimates from the combined reference population: assuming homogenous SNP effects among all breeds using a “joint-breed approach” or assuming different SNP effects for each of the purebreds using a “BOA approach.” Thus, the joint-breed approach treated all populations in the combined reference population as one breed, whereas the BOA approach treated all populations in the reference as different breeds. In predicting genetic merit for crossbred animals using the BOA approach, alleles of each SNP were traced back to its breed of origin, and the SNP effect from the respective breed was used. See detail of these approaches in Table 2.

**Table 2:** Prediction accuracies and biases (in parenthesis) for Danish Holstein (HOL), Danish Jersey (JER), Swedish Red (RDC), three-breed rotational crosses (MIX), and JER × HOL rotational crosses (JXH) using individual breeds, all purebreds HOL, JER, and RDC, or all breeds in the reference population.

Reference population^a^						Test population^b^				
HOL	RDC	JER	MIX	JXH	Model	HOL	RDC	JER	MIX	JXH
F	–	–	–	–	Within breed	0.764_e_ (0.933)	0.149_g_ (0.457)	0.068_h_ (0.249)	0.546_f_ (0.895)	0.595_f_ (0.902)
–	F	–	–	–	Within breed	0.120_h_ (0.386)	0.739_e_ (0.998)	0.075_h_ (0.294)	0.362_h_ (0.782)	0.117_i_ (0.379)
–	–	F	–	–	Within breed	0.017_i_ (0.131)	0.056_i_ (0.340)	0.644_e_ (1.011)	0.147_i_ (0.556)	0.262_h_ (0.706)
–	–	–	F	–	Within breed	0.479_g_ (0.940)	0.542_f_ (0.979)	0.513_g_ (0.971)	0.559_f_ (0.932)	0.537_g_ (0.938)
–	–	–	–	F	Within breed	0.549_f_ (0.914)	0.105_h_ (0.348)	0.627_f_ (0.991)	0.473_g_ (0.830)	0.662_e_ (0.939)
F	F	F	–	–	Pure-BOA	–	–	–	0.663_e_ (0.865)	0.656_e_ (0.840)
F	F	F	F	F	Joint breed	0.791_d_ (1.009)	0.754_d_ (0.976)	0.734_d_ (1.037)	0.764_c_ (0.962)	0.792_c_ (0.967)
F	F	F	F	F	BOA	0.798_c_ (1.035)	0.761_c_ (1.021)	0.743_c_ (1.083)	0.753_d_ (0.927)	0.789_d_ (0.954)

^a^F = full genotype and phenotype information.

^b^Standard errors within test populations HOL, RDC, MIX, and JXH were similar and between 0.004 and 0.018 across reference populations. For the JER test population, they were between 0.014 and 0.023 across reference populations.

^c–i^Accuracies within test population with no common subscripts differ significantly (*p <* 0.05).

Genetic merits for pure breeds in the within-breed, joint-breed, and BOA approaches were predicted by multiplying SNP effects with the corresponding allele counts. For prediction of GMs in crossbred animals, the same procedures were used, with the addition of fixed breed effects multiplied by genomic breed proportions for joint-breed and BOA models. In the pure-BOA, the breed-origin of alleles for crossbred animals were considered in predictions as in BOA approach, but only the SNP effects from individual pure breed analysis were used, and a fixed breed effects multiplied by genomic breed was added.

Samples of each parameter were obtained by sampling from their full-conditional posterior distribution using the Markov-chain Monte Carlo algorithm. The chain length for the analyses was 50,000 cycles, the first 10,000 of which were discarded as burn-in. Every tenth sample from the post burn-in cycles was saved for posterior analysis, resulting in 4000 posterior samples. Each parameter’s estimate was based on the mean value of the posterior samples. All of the analyses were carried out in Julia using self-written scripts.

Each scenario was replicated 25 times and the average of prediction accuracies across all replicates are given as the results. The prediction accuracies were calculated as the correlation between the simulated true genotypic value and predicted GM of the test animals. The prediction accuracies were compared between relevant scenarios by a paired t-test using a significance level of *p* < 0.05.

### Genomic prediction in three-breed crosses with different sire breeds

Three alternative test populations were created to investigate the importance of sire breed in the MIX animals: HOL-sired MIX animals from generation 12, RDC-sired MIX animals from generation 11, and JER-sired MIX animals from generation 10. The reference populations were the three previous generations, i.e., generation 9, 10, and 11 for HOL-sired MIX animals and accordingly generations 8–10 and 7–9 for RDC-sired and JER-sired MIX animals, respectively. To avoid different breed compositions in the reference populations, JXH animals were left out of the combined reference population. Thus, the average GBP was the same regardless of which generations were used in the reference. The GBP in the combined reference population (without JXH) was 0.416 HOL and RDC, and 0.168 JER. The three test populations had genomic predictions obtained from eight different compositions of reference populations using the BOA approach. The reference populations were all including MIX, and included all, none, one, or two of the pure breeds.

### Genomic prediction with different numbers of crossbreds in the reference population

We investigated the effect of reference population size of MIX and JXH and the difference between selecting dam lines for the reference population and selecting animals at random. The reference population included the three pure breeds, MIX and JXH. In four different scenarios, we selected 1000, 500, 250, and 100 of each MIX and JXH animals from generation 11 and their dams and grand dams from generations 10 and 9, respectively. In two additional scenarios, 500 and 100 animals from each of MIX and JXH were selected randomly each of the three generations (9, 10, 11) for the reference population. These analyses were made using the BOA approach.

### Genomic prediction using summary statistics

We investigated cases of reference populations with different combinations of information sources. These combinations were full genotype and phenotype information was available from all pure breeds, only on HOL, only HOL and RDC, or only HOL and JER. In the cases where full information was unavailable for some of the breeds, those breeds either did not contribute any information or contributed summary statistics. The last two scenarios were the cases where all breeds contributed with summary statistics or did not contribute any information. In all nine scenarios, full information from MIX and JXH was available in the reference population. These scenarios were analyzed using the BOA approach. Additionally, we analyzed these scenarios using the joint-breed approach, results of which are presented in Supplementary File 2.

## Results

### Genomic prediction using different reference populations and models

Using the within-breed approach yielded higher prediction accuracies using own reference (i.e., using the reference population of the same breed) than across breeds (i.e., using a reference population of another breed) (Table 2). There were significant differences (*p* < 0.05) in prediction accuracies between the reference populations used within most of the test populations. For prediction of JER the difference between accuracies using HOL (0.068) and RDC (0.075) reference populations was not significant. The difference between predicting MIX using HOL (0.546) and MIX (0.559) reference populations was also not significant. Predictions were generally more biased when based on a reference population of another breed.

The highest prediction accuracy was achieved for any of the five test populations when using a combined reference population that included animals from all five populations (Joint-breed or BOA) (Table 2). JER and the two crossbred test populations, MIX and JXH, gained the most by using a combined reference population compared with using their own reference population by the within-breed models. For the purebred test populations (HOL, RDC, and JER), the BOA approach was slightly better (*p* < 0.05) than the joint-breed approach, whereas it was vice versa for MIX and JXH (*p* < 0.05). For the Pure-BOA models, predictions of MIX and JXH were more biased than when MIX or JXH were used as reference populations in within-breed predictions. The Joint-breed and BOA models provided less biased predictions than the Pure-BOA model.

### Genomic prediction using summary statistics

Having full information available (full scenario) from all the breeds was better than having only the summary statistics from the pure breeds or having no pure breed information available (Table 3). The difference between full scenario and summary statistics for all purebreds was much smaller than differences between summary statistics and no information from the purebreds. The prediction accuracies for JER and RDC decreased greatly when reference population changed from the full scenario to the scenario where summary statistics or no information was available from both of those breeds and full information was available from HOL. When only summary statistics from the respective pure breeds were available, the decrease in prediction accuracy was largest for the RDC population (from 0.761 in the full scenario to 0.553. However, when no information was available from the respective pure breeds, the JER test population was affected the most (from 0.743 in the full scenario to 0.499).

**Table 3:** Prediction accuracies and biases (in parenthesis) for Danish Holstein (HOL), Danish Jersey (JER), Swedish Red (RDC), three-breed rotational crosses (MIX) and JER × HOL rotational crosses (JXH) in different scenarios including full phenotype and genotype data (F), summary statistics (S), or no information (-), using the BOA approach.

Data from reference population^a^					Test population^b^				
HOL	RDC	JER	MIX	JXH	HOL	RDC	JER	MIX	JXH
F	F	F	F	F	0.798_c_ (1.035)	0.761_d_ (1.021)	0.743_d_ (1.083)	0.753_d_ (0.927)	0.789_d_ (0.954)
S	S	S	F	F	0.752_e_ (1.142)	0.731_e_ (1.252)	0.710_e_ (1.229)	0.720_e_ (1.023)	0.768_e_ (1.068)
F	S	S	F	F	0.795_d_ (0.837)	0.554_f_ (1.068)	0.673_f_ (1.045)	0.718_e_ (0.816)	0.782_g_ (0.833)
F	S	F	F	F	0.795_d_ (0.838)	0.553_f_ (1.073)	0.748_c_ (0.960)	0.718_e_ (0.807)	0.785_f_ (0.816)
F	F	S	F	F	0.799_c_ (1.033)	0.762_c_ (1.017)	0.713_e_ (1.050)	0.755_c_ (0.930)	0.791_c_ (0.959)
–	–	–	F	F	0.605_f_ (1.408)	0.537_g_ (2.169)	0.657_g_ (1.290)	0.590_h_ (1.104)	0.676_ij_ (1.162)
F	–	–	F	F	0.755_e_ (1.443)	0.528_h_ (2.806)	0.491_i_ (1.953)	0.647_g_ (1.208)	0.669_j_ (1.203)
F	–	F	F	F	0.759_e_ (1.357)	0.529_h_ (2.530)	0.667_fg_ (1.590)	0.655_f_ (1.143)	0.693_h_ (1.133)
F	F	–	F	F	0.763_e_ (1.315)	0.743_e_ (1.330)	0.499_h_ (1.786)	0.706_e_ (1.126)	0.679_i_ (1.143)

^a^F = full genotype and phenotype information; S = summary statistics available on genotype and phenotype information.

^b^Standard errors within test populations HOL, RDC, MIX, and JXH were similar and between 0.004 and 0.017 across reference populations. For the JER test population, they were between 0.013 and 0.017 across reference populations.

^c–i^Accuracies within test population with no common subscripts differ significantly (*p <* 0.05).

### Genomic prediction in three-breed crosses with different sire breeds

As shown in Tale 4, when full information was available from all the pure breeds, the HOL-sired crossbred animals had significantly higher prediction accuracy (0.731) than RDC-sired (0.709) and JER-sired crossbred animals (0.672. Interestingly, the prediction accuracy was significantly highest for JER-sired animals (0.509) when no information was available from the pure breeds. The prediction accuracies were positively affected when information from the same pure breed reference as the sire breed of the MIX test population was available.

### Genomic prediction with different numbers of crossbreds in the reference population

The number of MIX and JXH animals in the reference population played a significant role in prediction accuracy, regardless of the test population (Table 5). The prediction accuracies decreased as the number of MIX and JXH animals in the reference population decreased, especially for JER, MIX, and JXH. Except for the MIX population, there was a significantly favorable effect of selecting 1500 + 1500 MIX and JXH animals based on daughter–dam relationship in the reference population. However, it was only for the JXH test population where it was significant to select 300 + 300 MIX and JXH (0.680) based on daughter–dam relationships rather than at random (0.667).

## Discussion

### Summary statistics for multibreed genomic prediction

Our results showed that it is essential to involve all breeds in the crossbreeding system when forming the reference populations, to gain higher prediction accuracies. Moreover, including crossbred animals in the reference population will benefit the genomic prediction of crossbreds (and purebreds) even more. However, having genotype and phenotype data available from all pure breeds in a reference population is not always feasible. Therefore, we investigated genomic prediction of crossbred animals using a BOA approach with two kinds of information from the reference population: (i) full information from crossbred and purebred animals, and (ii) full information from crossbred animals but summary statistics from all or only some of the pure breeds.

When using summary statistics from all three pure breeds, the prediction accuracies were only slightly lower than for the full scenario, but much higher than for the scenario using no information from the pure breeds. The differences were most prominent for the HOL and RDC test populations and smallest for the JER and JXH test populations. Those differences are probably due to different breed compositions in the reference populations and the genetic relationship between reference and test populations. When only JXH and MIX animals were included in the reference population, the proportion of JER genes would be 0.44 compared with 0.26 in the scenario when the three pure breeds were included as well. Hence, the genetic relationship with the reference population would on average be closer for JER and JXH test populations, than for HOL and RDC when only MIX and JXH was included in the reference population.

For JXH, having full information available from HOL in the reference population and either summary statistics from RDC or JER or both seemed sufficient to reach the prediction accuracies in the full scenario. This was likely because all JXH animals in the test population were HOL-sired, did not share genetic relationship with RDC, and the added JER reference population was small. If the JXH test animals were all JER-sired, having full information from JER, but not HOL, in the reference might not be sufficient because the size of the JER reference was much smaller than HOL. However, at small population sizes, the effective population size (*N*_*e*_) is usually small and thus there are fewer effective chromosome segments to predict, compared with large populations with high *N*_*e*_ (Meuwissen 2009). Thus, the JER reference population might not need to increase to the same size as HOL, for JER-sired JXH to achieve prediction accuracies as high as HOL-sired JXH.

In livestock species, the potential of using summary statistics from multiple populations is little exploited for genomic prediction, partially because trait definitions, genomic prediction models, and variance components tend to differ between populations (Minozzi et al. 2012). In human genetics, however, the use of genome-wide association summary statistics has become a common approach to enhance risk assessments of complex traits and diseases (Spiliopoulou et al. 2015; Maier et al. 2018; Allegrini et al. 2019).

The exchange of semen and embryos internationally has been done for decades, making it possible to develop crossbreeding using foreign breeds. However, due to logistics or political barriers (Tenopir et al. 2011), the exchange of genotype and phenotype data from an entire breed population between countries is not always possible. Therefore, genomic prediction in crossbred animals can become difficult if foreign breeds are involved. Using summary statistics only requires sharing summarized data but not full datasets of genotypes and phenotypes. Furthermore, using summary statistics makes it more practically feasible to run prediction models based on multiple populations because it does not require a large amount of data. A base for sharing summary statistics on dairy cattle breed populations between countries is under development in the Interbull SNPMace project (Jighly et al. 2019). However, the SNPMace effort currently only accommodates combining data from different populations of the same breed but not crossbreeding.

The SNPMace model includes prediction error co-variances (PEC) between SNPs (Jighly et al. 2019), whereas our model ignores the correlation of prediction errors between SNPs and only considers PEV of estimated SNP effects. Thus, the SNPMace model may be more accurate than our model for summary statistics. However, complete summary statistic information, including PEC between SNPs, may not always be possible to achieve, and in that case, even approximate integration using PEV may improve prediction accuracies (Vandenplas et al. 2018).

### Small breed populations and breed proportions in the reference population

The current study showed that a small breed population (JER) gains more from a multibreed reference population than a large breed population (HOL and RDC). That is in accordance with other studies (Erbe et al. 2012; Olson et al. 2012; Hozé et al. 2014). However, the test breed need to be somewhat closely related to the breeds in the multibreed reference to gain from it (Brøndum et al. 2011). Therefore, including “any” breed in multibreed references and assuming equal SNP effects may not always result in higher prediction accuracy compared with within-breed predictions, at least not for all traits, and can even cause a loss in prediction accuracy. Adding crossbred data to the combined reference population increases the data size, which may help improve the accuracies for small pure-breed populations (Veroneze et al. 2014). In a simulation study, Karaman et al. (2021) showed that prediction accuracies increased much more for JER than HOL when adding crossbreds to a combined reference population. Thus, adding crossbreds to the reference could benefit the small breed population, because the marginal value for each added data is higher for the small breed population than for the large breed population.

Many previous studies discuss an issue of unbalanced breed proportions in a combined reference population, causing the breed with the most contribution to dominate the SNP effects (Olson et al. 2012; van den Berg et al. 2020; Khansefid et al. 2020). In our study, JER contributed relatively little to the combined reference population compared with HOL and RDC, which caused prediction accuracies to be noticeable smaller for JER than for HOL and RDC when using the combined reference population. The use of BOA approach instead of the joint-breed approach can eliminate the unfavorable effect of unbalanced breed proportions in the reference population, and thus benefit the small breed. This probably explains why JER benefitted more from the BOA approach than the joint-breed approach (Table 2). Additionally, it was evident that including all pure breeds in the reference population had a significantly higher effect on HOL-sired MIX than JER-sired MIX (Table 4).

**Table 4:** Prediction accuracies and biases (in parenthesis) for Danish Holstein-sired (MIX_HOL_), Danish Jersey-sired (MIX_JER_), and Swedish Red-sired (MIX_RDC_) three-breed rotational crosses estimated on full genotype and phenotype information (F) or no information (-) from the respective pure breeds and MIX in the reference population.

Data from reference population^h^				Test population^i^		
HOL	RDC	JER	MIX	MIX_HOL_	MIX_RDC_	MIX_JER_
F	F	F	F	_a_0.731^a^ (0.912)	_a_0.709^b^ (0.875)	_a_0.672^c^ (0.902)
–	–	–	F	_h_0.433^c^ (1.267)	_h_0.458^b^ (1.211)	_g_0.509^a^ (1.586)
F	–	–	F	_d_0.647^a^ (1.208)	_f_0.516^c^ (1.088)	_e_0.574^b^ (1.251)
–	F	–	F	_f_0.542^b^ (1.184)	_d_0.655^a^ (1.189)	_f_0.543^b^ (1.320)
–	–	F	F	_g_0.460^c^ (1.164)	_g_0.488^b^ (1.094)	_d_0.594^a^ (1.406)
–	F	F	F	_e_0.550^c^ (1.112)	_c_0.660^a^ (1.096)	_c_0.611^b^ (1.221)
F	–	F	F	_c_0.655^a^ (1.143)	_e_0.532^b^ (1.023)	_b_0.639^a^ (1.180)
F	F	–	F	_b_0.706^a^ (1.126)	_b_0.684^b^ (1.091)	_d_0.595^c^ (1.140)

^a–g^ Accuracies within test population (column) with different subscripts and accuracies within reference population (row) with different superscripts differ significantly (p < 0.05).

^h^ F = full genotype and phenotype information.

^i^ Standard errors within test populations were similar and between 0.007 and 0.013 across reference populations.

### The pure-BOA, BOA, and joint-breed approaches

The purpose of the pure-BOA approach in the current study was to evaluate an improved version of the method used by VanRaden et al. (2020). The main difference from that study was that we used breed-specific SNP effects per SNP marker by tracing back each allele to the breed of origin instead of using marker effects of all breeds and weigh them by a single breed proportion (adjusted GBP; base breed proportions; BBR, VanRaden et al. 2020) for all markers.

Because of the close genetic relationship between breeds (0.62–0.87) in our study, we did not see a considerable difference in prediction accuracy using the joint versus the BOA approach. If breeds are highly correlated, the amount of unique SNP effects from individual breeds will be small, and prediction accuracies, particularly for the small breed, will be positively affected when using a joint-breed approach (Karaman et al. 2021). However, if breeds are genetically distant, assuming homogeneous SNP effects may neutralize unique SNP effects and cause low prediction accuracies for the individual test populations. Instead, considering different SNP effects and tracing each allele back to the breed of origin will capture SNP effects that were neutralized in the joint-breed approach. Furthermore, tracing the breed-origin to allele is easier when breeds are unrelated rather than closely related (Vandenplas et al. 2016). Thus, using a BOA approach (versus a joint-breed approach) is most beneficial if the breeds are genetically distant (Sevillano et al. 2017; Karaman et al. 2021).

### Construction of reference population

Except for the simulation scenarios in Table 5, we assumed that the crossbred reference populations (MIX and JXH) would be as large as the HOL and RDC reference populations (3150 animals). This assumption is probably optimistic since the number of (systematically) crossbred animals is small in most dairy cattle populations nowadays, and the number of genotyped crossbreds is even smaller. When the number of crossbred animals was reduced in the combined reference population (Table 5), the prediction accuracy decreased significantly for all test populations, primarily for MIX, JXH, and JER. This was probably because the proportion of JER-genes decreased from 25% when 3000 of each MIX and JXH were included, to 12% when only 300 were included. The proportions of HOL and RDC did increase from 42 to 45% and 32 to 43%, respectively, when decreasing the number of JXH and MIX from 3000 to 300. In addition, the relationship between animals in the reference and the MIX and JXH test populations decreased because not all dams of the test animals were in the reference.

**Table 5:** Prediction accuracies and biases (in parenthesis) for Danish Holstein (HOL), Danish Jersey (JER), Swedish Red (RDC), three-breed rotational crosses (MIX), and JER × HOL rotational crosses (JXH) with various numbers of MIX and JXH in the reference population. Full phenotype and genotype information from all pure breed populations was used in the reference population.

	Test population^h^				
No. of MIX + JXH in the reference	HOL	RDC	JER	MIX	JXH
3000 + 3000 (all)	0.797_a_ (1.035)	0.759_a_ (1.020)	0.742_a_ (1.083)	0.751_a_ (0.926)	0.787_a_ (0.953)
1500 + 1500 (selected)	0.783_b_ (1.032)	0.752_b_ (1.026)	0.712_b_ (1.085)	0.713_b_ (0.882)	0.749_b_ (0.922)
1500 + 1500 (random)	0.786_c_ (1.035)	0.747_c_ (1.034)	0.702_c_ (1.082)	0.709_b_ (0.885)	0.736_c_ (0.905)
750 + 750 (selected)	0.776_d_ (1.022)	0.745_c_ (1.017)	0.689_d_ (1.059)	0.681_c_ (0.828)	0.717_d_ (0.880)
300 + 300 (selected)	0.769_e_ (1.013)	0.742_d_ (1.010)	0.665_e_ (1.044)	0.649_d_ (0.781)	0.680_e_ (0.829)
300 + 300 (random)	0.770_e_ (1.010)	0.742_d_ (1.011)	0.658_e_ (1.023)	0.649_d_ (0.776)	0.667_f_ (0.801)

^a-g^Accuracies within test population with different subscripts differ significantly (*p* < 0.05).

^h^Standard errors within test populations HOL, RDC, MIX, and JXH were similar and between 0.005 and 0.009 across reference populations. For the JER test population, they were between 0.014 and 0.015 across reference populations.

The genetic relationship between animals in the test and reference populations was, in previous studies, shown to be of importance in genomic prediction (Habier et al. 2007; Pszczola et al. 2012; Clark et al. 2012)—also in the setting of multibreed genomic prediction (Erbe et al. 2012; Hozé et al. 2014). A strategy to increase genomic prediction accuracies is to include animals in the reference population that are related to each other (e.g., sire lines or dam lines) and to the animals in the test population. For example, combining reference populations of Holstein, Montbéliarde, and Normande, Hozé et al. (2014) investigated the effects of animal relationships between test and reference populations (bulls only). For within-breed predictions, prediction accuracies were considerably higher if sires of the test animals were in the reference population. However, for multibreed predictions, the prediction accuracies did not differ notably for animals with sires in the reference population, while it nearly doubled for those without. Erbe et al. (2012) also found higher prediction accuracies for animals with a sire included in the reference, but the increase was smaller since the reference population was twice as large as in Hozé et al. (2014). For small reference populations, the effect of family relationships has a higher influence on the accuracy of genomic prediction than the effect of LD, compared with large reference population (Clark et al. 2012). In our study, the effects of selecting the reference at random or based on the daughter–dam relationship on prediction accuracy were different between JXH and MIX test populations. For the JXH test population, selected dam lines increased prediction accuracy significantly, but only slightly for MIX. However, the differences were small, probably because the relationship between the animals within the reference population was similar regardless of selection within the JXH and MIX animals. In the 1500 + 1500 scenarios, about 30% of the animals in the reference were MIX and JXH, while it was only 8% in the 300 + 300 scenarios. Therefore, the prediction accuracy for MIX tended to be higher when dams were selected in the 1500 + 1500 versus picked at random, but not in the 300 + 300 scenario. Both Pérez-Cabal et al. (2010) and Pszczola et al. (2012) found that, depending on how strictly they were selected, the relationship between sires selected for the reference population was the same or even slightly lower than if the sires in the reference were selected at random.

### Limitations in the simulations

A primary reason for crossbreeding is the benefits of heterosis effects, which we ignored in the simulation of phenotypes and estimation of GMs in this study. Heterosis effects are caused by non-additive effects due to dominance between alleles at the same loci (Wittenburg et al. 2011; Su et al. 2012). In dairy cattle, heterosis effects tend to favor the most economically important traits (Sørensen et al. 2008; Jönsson 2015; Clasen et al. 2017). If we had included heterosis effects in the simulation of phenotypes, the prediction accuracies for GM in MIX and JXH would be lower than estimated in this study, because the prediction models did not account fully for non-additive effects. Ignoring non-additive effects, such as dominance effects, in genomic prediction can increase the unbiasedness of genomic prediction (e.g., Wittenburg et al. 2011; Su et al. 2012; Esfandyari et al. 2016). Thus, non-additive effects should be considered if we apply the current models for predicting GMs on real animals.

For computational reasons, we simulated only the five first chromosomes, which is barely a quarter of the bovine genome. Genomic prediction accuracy for within-breed predictions can be approximated by knowing the size of the reference population, *N*_*e*_, the heritability of the trait, and the length of the genome (Daetwyler et al. 2008; Meuwissen 2009; Goddard 2009). To obtain the same prediction accuracies for an increased length of the genome, by including more chromosomes, requires a relative increase in the number of animals in the reference population, if the heritability and *N*_*e*_ remain constant (Meuwissen 2009). Furthermore, the approximations need to be corrected for the genetic correlation between the reference breed and the test breed, which may require a larger reference population the smaller the genetic correlation between the breeds is (Wientjes et al. 2015). The results from the within-breed approach in this study are still valid if the predictions were scaled up to the whole genome, and the size of the reference population is increased according to the genetic relationship between breeds. The BOA approach has the advantage of using individual within-breed data and enhancing the size of the reference population by including crossbred animals.

In this paper, using the joint approach, although the same weight was implicitly given to the summary statistics derived either from a large breed (HOL and RDC) or a small breed (JER), the differences in accuracy of the estimated SNP effects from the within-breed analyses are accounted for by PEVs. Similarly, when using BOA with summary statistics, the per-breed accuracies of the estimated SNP effects are also accounted for by their corresponding PEVs.

In this work, we assumed that breed origin of each allele is known without error. In real life applications, those need to be estimated from the data, and several steps to achieve this may challenge BOA models and may limit the benefit from BOA models (Guillenea et al. 2022).

## Conclusions

In this study, we found a potential of using summary statistics for genomic prediction in rotationally crossbred dairy cattle using a multibreed reference population approach and tracing alleles back to the breed of origin. Combining pure breeds and crossbreds in a single reference population yielded higher prediction accuracies for both purebred and crossbred animals. The combined reference population was also beneficial for the smallest breed population in this study.

## Supplementary information

Supplementary File 2

Supplementary File 1

## Data Availability

Haplotype data for the base populations of Holstein, Red Dairy Cattle, and Jersey can be downloaded at: https://figshare.com/articles/dataset/Base_population_data/17198534.
