# Supplementary material for: Multibreed genomic prediction using summary statistics and a breed-origin-of-alleles approach
Source: Heredity (Edinb). 2023 May 25;131(1):33–42. doi: 10.1038/s41437-023-00619-4 (PMC10313778; doi:10.1038/s41437-023-00619-4)
Supplement: Supplementary file 1 — Supplementary File 2 [file 41437_2023_619_MOESM1_ESM.docx]

**Supplementary File 2.** Prediction accuracies and biases (in parenthesis) for Holstein (HOL), Danish Jersey (JER), Swedish Red (RDC), three-breed rotational crosses (MIX) and Jersey x Holstein rotational crosses (JXH) based on a combined reference population using a joint model

| Data from reference population^1^ | | | | | Test population^2^ | | | | |
| --- | --- | --- | --- | --- | --- | --- | --- | --- | --- |
| HOL | RDC | JER | MIX | JXH | HOL | RDC | JER | MIX | JXH |
| F | F | F | F | F | 0.791_bc_ (1.009) | 0.754_b_ (0.976) | 0.734_a_ (1.037) | 0.764_a_ (0.962) | 0.792_b_ (0.967) |
| S | S | S | F | F | 0.738_d_ (1.060) | 0.704_cd_ (1.042) | 0.701_d_ (1.090) | 0.723_d_ (1.015) | 0.766_c_ (1.040) |
| F | S | S | F | F | 0.789_c_ (1.023) | 0.703_d_ (0.875) | 0.706_c_ (0.976) | 0.750_b_ (0.939) | 0.792_b_ (0.976) |
| F | S | F | F | F | 0.789_c_ (1.021) | 0.706_c_ (0.870) | 0.732_b_ (1.054) | 0.750_b_ (0.937) | 0.791_b_ (0.981) |
| F | F | S | F | F | 0.791_bc_ (1.015) | 0.754_b_ (0.982) | 0.706_c_ (0.957) | 0.764_a_ (0.966) | 0.792_b_ (0.965) |
| - | - | - | F | F | 0.621_e_ (0.951) | 0.539_e_ (0.918) | 0.669_e_ (1.002) | 0.643_e_ (0.928) | 0.708_d_ (0.963) |
| F | - | - | F | F | 0.794_a_ (1.015) | 0.541_e_ (0.876) | 0.666_ef_ (0.985) | 0.730_c_ (0.954) | 0.793_ab_ (0.978) |
| F | - | F | F | F | 0.792_b_ (1.012) | 0.541_e_ (0.869) | 0.737_a_ (1.042) | 0.729_cd_ (0.947) | 0.794_a_ (0.972) |
| F | F | - | F | F | 0.792_b_ (1.011) | 0.755_a_ (0.980) | 0.663_f_ (0.977) | 0.764_a_ (0.967) | 0.790_b_ (0.973) |

^a-g^ Accuracies within test population with no common subscript differ significantly (p < 0.05)

^1^F = full genotype and phenotype information; S = summary statistics available on genotype and phenotype information­

^2^Standard errors within test populations HOL, RDC, MIX, and JXH were similar and between 0.004 and 0.011 across reference populations. For the JER test population they were between 0.014 and 0.018 across reference populations.
