# Supplementary material for: Multibreed genomic prediction using summary statistics and a breed-origin-of-alleles approach
Source: Heredity (Edinb). 2023 May 25;131(1):33–42. doi: 10.1038/s41437-023-00619-4 (PMC10313778; doi:10.1038/s41437-023-00619-4)
Supplement: Supplementary file 2 — Supplementary File 1 [file 41437_2023_619_MOESM2_ESM.pdf]

Supplementary File 1: Multi-breed genomic prediction using summary statistics, and augmenting data with admixed individuals

In this section, we present briefly the derivations that paved the way to the integration of summary statistics from pure breed analysis to the data analysis of admixed individuals using BOA models. We start with revisiting the necessary derivations for a joint analysis of multi-breed data using summary statistics, which was already detailed in Vandenplas et al. [1]. Then, we present the derivations for the BOA model assuming uncorrelated SNP effects between the breeds [2].

1 Data augmentation and joint analysis

Assume the following model for the separate analysis of each single-breed ($i = A, B, C$), where there are n_i animals in each.

$$\mathbf{y}_i = \mathbf{1}\mu_i + \mathbf{M}_i\boldsymbol{\beta}_i + \mathbf{e}_i$$

where $\mathbf{y}_i$ is the vector of phenotypic records, μ_i is the general mean, $\mathbf{M}_i$ is the matrix of genotypes coded as 0,1 or 2, $\boldsymbol{\beta}_i$ is the vector of SNP effects assumed to follow a normal distribution as $\boldsymbol{\beta}_i \sim N(\mathbf{0}, \mathbf{B}_i)$ where $\mathbf{B}_i$ is usually a digaonal matrix of $\mathbf{I}\sigma^2_{\beta_i}$, and $\mathbf{e}_i$ is the vector of residuals assumed to follow a normal distribution as $\mathbf{e}_i \sim N\left(\mathbf{0}, \mathbf{D}_i\sigma^2_{e_i}\right)$, for breed i. Then, one needs to solve the following set of equations for the analysis of data from breed i:

$$\begin{bmatrix} \mathbf{1}'\mathbf{D}_i^{-1}\sigma_{e_i}^{-2}\mathbf{1} & \mathbf{1}'\mathbf{D}_i^{-1}\sigma_{e_i}^{-2}\mathbf{M}_i \\ \mathbf{M}_i'\mathbf{D}_i^{-1}\sigma_{e_i}^{-2}\mathbf{1} & \mathbf{M}_i'\mathbf{D}_i^{-1}\sigma_{e_i}^{-2}\mathbf{M}_i + \mathbf{B}_i^{-1} \end{bmatrix} \begin{bmatrix} \tilde{\mu}_i \\ \tilde{\boldsymbol{\beta}}_i \end{bmatrix} = \begin{bmatrix} \mathbf{1}'\mathbf{D}_i^{-1}\sigma_{e_i}^{-2}\mathbf{y}_i \\ \mathbf{M}_i'\mathbf{D}_i^{-1}\sigma_{e_i}^{-2}\mathbf{y}_i \end{bmatrix}$$

It follows that,

$$\tilde{\mu}_i = \left(\mathbf{1}'\mathbf{D}_i^{-1}\sigma_{e_i}^{-2}\mathbf{1}\right)^{-1}\left(\mathbf{1}'\mathbf{D}_i^{-1}\sigma_{e_i}^{-2}\mathbf{y}_i - \mathbf{1}'\mathbf{D}_i^{-1}\sigma_{e_i}^{-2}\mathbf{M}_i\tilde{\boldsymbol{\beta}}_i\right) \tag{1}$$

$$\left(\mathbf{M}_i'\mathbf{D}_i^{-1}\sigma_{e_i}^{-2}\mathbf{1}\right)\tilde{\mu}_i + \left(\mathbf{M}_i'\mathbf{D}_i^{-1}\sigma_{e_i}^{-2}\mathbf{M}_i + \mathbf{B}_i^{-1}\right)\tilde{\boldsymbol{\beta}}_i = \mathbf{M}_i'\mathbf{D}_i^{-1}\sigma_{e_i}^{-2}\mathbf{y}_i \tag{2}$$

inserting 1 into 2 we get:

$$\begin{aligned} \left(\mathbf{M}_i'\mathbf{D}_i^{-1}\sigma_{e_i}^{-2}\mathbf{1}\right)\left(\mathbf{1}'\mathbf{D}_i^{-1}\sigma_{e_i}^{-2}\mathbf{1}\right)^{-1}\left(\mathbf{1}'\mathbf{D}_i^{-1}\sigma_{e_i}^{-2}\mathbf{y}_i - \mathbf{1}'\mathbf{D}_i^{-1}\sigma_{e_i}^{-2}\mathbf{M}_i\tilde{\boldsymbol{\beta}}_i\right) + \left(\mathbf{M}_i'\mathbf{D}_i^{-1}\sigma_{e_i}^{-2}\mathbf{M}_i + \mathbf{B}_i^{-1}\right)\tilde{\boldsymbol{\beta}}_i &= \mathbf{M}_i'\mathbf{D}_i^{-1}\sigma_{e_i}^{-2}\mathbf{y}_i \\ \left(\mathbf{M}_i'\mathbf{D}_i^{-1}\sigma_{e_i}^{-2}\mathbf{M}_i + \mathbf{B}_i^{-1}\right)\tilde{\boldsymbol{\beta}}_i - \left(\mathbf{M}_i'\mathbf{D}_i^{-1}\sigma_{e_i}^{-2}\mathbf{1}\right)\left(\mathbf{1}'\mathbf{D}_i^{-1}\sigma_{e_i}^{-2}\mathbf{1}\right)^{-1}\left(\mathbf{1}'\mathbf{D}_i^{-1}\sigma_{e_i}^{-2}\mathbf{M}_i\right)\tilde{\boldsymbol{\beta}}_i &= \mathbf{M}_i'\mathbf{D}_i^{-1}\sigma_{e_i}^{-2}\mathbf{y}_i - \left(\mathbf{M}_i'\mathbf{D}_i^{-1}\sigma_{e_i}^{-2}\mathbf{1}\right)\left(\mathbf{1}'\mathbf{D}_i^{-1}\sigma_{e_i}^{-2}\mathbf{1}\right)^{-1}\left(\mathbf{1}'\mathbf{D}_i^{-1}\sigma_{e_i}^{-2}\mathbf{y}_i\right) \\ \left[\mathbf{M}_i'\left(\mathbf{D}_i^{-1}\sigma_{e_i}^{-2} - \left(\mathbf{D}_i^{-1}\sigma_{e_i}^{-2}\mathbf{1}\right)\left(\mathbf{1}'\mathbf{D}_i^{-1}\sigma_{e_i}^{-2}\mathbf{1}\right)^{-1}\mathbf{1}'\mathbf{D}_i^{-1}\sigma_{e_i}^{-2}\right)\mathbf{M}_i + \mathbf{B}_i^{-1}\right]\tilde{\boldsymbol{\beta}}_i &= \mathbf{M}_i'\left(\mathbf{D}_i^{-1}\sigma_{e_i}^{-2} - \left(\mathbf{D}_i^{-1}\sigma_{e_i}^{-2}\mathbf{1}\right)\left(\mathbf{1}'\mathbf{D}_i^{-1}\sigma_{e_i}^{-2}\mathbf{1}\right)^{-1}\mathbf{1}'\mathbf{D}_i^{-1}\sigma_{e_i}^{-2}\right)\mathbf{y}_i \end{aligned}$$

The system of equations that needs to be solved for $\tilde{\boldsymbol{\beta}}_i$ is

$$\left[\mathbf{M}_i'\left(\mathbf{D}_i^{-1}\sigma_{e_i}^{-2} - \left(\mathbf{D}_i^{-1}\sigma_{e_i}^{-2}\mathbf{1}\right)\left(\mathbf{1}'\mathbf{D}_i^{-1}\sigma_{e_i}^{-2}\mathbf{1}\right)^{-1}\mathbf{1}'\mathbf{D}_i^{-1}\sigma_{e_i}^{-2}\right)\mathbf{M}_i + \mathbf{B}_i^{-1}\right]\tilde{\boldsymbol{\beta}}_i = \mathbf{M}_i'\left(\mathbf{D}_i^{-1}\sigma_{e_i}^{-2} - \left(\mathbf{D}_i^{-1}\sigma_{e_i}^{-2}\mathbf{1}\right)\left(\mathbf{1}'\mathbf{D}_i^{-1}\sigma_{e_i}^{-2}\mathbf{1}\right)^{-1}\mathbf{1}'\mathbf{D}_i^{-1}\sigma_{e_i}^{-2}\right)\mathbf{y}_i$$

Under the assumption of homogeneous residual variance, $\mathbf{D}_i = \mathbf{I}$, it can be seen that

$$\left[\mathbf{M}_i'\mathbf{M}_i\sigma_{e_i}^{-2} + \mathbf{B}_i^{-1}\right]\tilde{\boldsymbol{\beta}}_i \approx \mathbf{M}_i'\sigma_{e_i}^{-2}\mathbf{y}_i$$

$$PEC\left(\tilde{\boldsymbol{\beta}}_i\right)^{-1} \approx \left[\mathbf{M}_i'\mathbf{M}_i\sigma_{e_i}^{-2} + \mathbf{B}_i^{-1}\right] \tag{3}$$

Now suppose that we combine the data from the pure breeds, *i.e.* A, B and C, together with the data of n_a admixed individuals (a), which leads to analysis using all available data in a joint analysis (J). The following model can be used.

$$\mathbf{y}_J = \mathbf{1}\mu_J + \mathbf{X}\mathbf{b}_J + \mathbf{M}\boldsymbol{\beta}_J + \mathbf{e}_J$$

In the above model $\mathbf{y}_J$ is the vector of phenotypic records, μ_J is the general mean, $\mathbf{X}$ is the matrix of breed proportions, $\mathbf{b}_J$ is the vector of breed effects, $\mathbf{M}$ is the matrix of genotypes coded as 0,1 or 2, $\boldsymbol{\beta}_J$ is the vector of joint SNP effects using the full data with an assumed distribution of $\boldsymbol{\beta}_J \sim N(\mathbf{0}, \mathbf{B}_J)$ where $\mathbf{B}_J$ is usually a digaonal matrix of $\mathbf{I}\sigma_{\beta_J}^2$, and $\mathbf{e}$ is the vector of residuals with $\mathbf{e}_J \sim N\left(\mathbf{0}, \mathbf{D}\sigma_{e_J}^2\right)$ and $\mathbf{D} = \begin{bmatrix} \mathbf{D}_A & \mathbf{0} & \mathbf{0} & \mathbf{0} \\ \mathbf{0} & \mathbf{D}_B & \mathbf{0} & \mathbf{0} \\ \mathbf{0} & \mathbf{0} & \mathbf{D}_C & \mathbf{0} \\ \mathbf{0} & \mathbf{0} & \mathbf{0} & \mathbf{D}_a \end{bmatrix}$. Note that this model treats the data as it belongs to a single homogenous breed, and thereby estimates a single set of SNP effects. The above model can also be written explicitly as follows.

$$\begin{bmatrix} \mathbf{y}_A \\ \mathbf{y}_B \\ \mathbf{y}_C \\ \mathbf{y}_a \end{bmatrix} = \mathbf{1}\mu_J + \begin{bmatrix} \mathbf{X}_A \\ \mathbf{X}_B \\ \mathbf{X}_C \\ \mathbf{X}_a \end{bmatrix}\mathbf{b}_J + \begin{bmatrix} \mathbf{M}_A \\ \mathbf{M}_B \\ \mathbf{M}_C \\ \mathbf{M}_a \end{bmatrix}\boldsymbol{\beta}_J + \begin{bmatrix} \mathbf{e}_A \\ \mathbf{e}_B \\ \mathbf{e}_C \\ \mathbf{e}_a \end{bmatrix} \tag{4}$$

From equation (4), the system of equations that needs to be solved is

$$\begin{bmatrix} \mathbf{1}'\mathbf{D}^{-1}\mathbf{1}\sigma_{e_J}^{-2} & \mathbf{1}'\mathbf{D}_A^{-1}\mathbf{X}_A\sigma_{e_J}^{-2} + \ldots + \mathbf{1}'\mathbf{D}_a^{-1}\mathbf{X}_a\sigma_{e_J}^{-2} & \mathbf{1}'\mathbf{D}_A^{-1}\mathbf{M}_A\sigma_{e_J}^{-2} + \ldots + \mathbf{1}'\mathbf{D}_a^{-1}\mathbf{M}_a\sigma_{e_J}^{-2} \\ \mathbf{X}_A'\mathbf{D}^{-1}\mathbf{1}\sigma_{e_J}^{-2} + \ldots + \mathbf{X}_a'\mathbf{D}_a^{-1}\mathbf{1}\sigma_{e_J}^{-2} & \mathbf{X}_A'\mathbf{D}_A^{-1}\mathbf{X}_A\sigma_{e_J}^{-2} + \ldots + \mathbf{X}_a'\mathbf{D}_a^{-1}\mathbf{X}_a\sigma_{e_J}^{-2} & \mathbf{X}_A'\mathbf{D}_A^{-1}\mathbf{M}_A\sigma_{e_J}^{-2} + \ldots + \mathbf{X}_a'\mathbf{D}_a^{-1}\mathbf{M}_a\sigma_{e_J}^{-2} \\ \mathbf{M}_A'\mathbf{D}_A^{-1}\mathbf{1}\sigma_{e_J}^{-2} + \ldots + \mathbf{M}_a'\mathbf{D}_a^{-1}\mathbf{1}\sigma_{e_J}^{-2} & \mathbf{M}_A'\mathbf{D}_A^{-1}\mathbf{X}_A\sigma_{e_J}^{-2} + \ldots + \mathbf{M}_a'\mathbf{D}_a^{-1}\mathbf{X}_a\sigma_{e_J}^{-2} & \mathbf{M}_A'\mathbf{D}_A^{-1}\mathbf{M}_A\sigma_{e_J}^{-2} + \ldots + \mathbf{M}_a'\mathbf{D}_a^{-1}\mathbf{M}_a\sigma_{e_J}^{-2} + \mathbf{B}_J^{-1} \end{bmatrix} \begin{bmatrix} \tilde{\mu}_J \\ \tilde{\mathbf{b}}_J \\ \tilde{\boldsymbol{\beta}}_J \end{bmatrix}$$

$$= \begin{bmatrix} \mathbf{1}'\mathbf{D}_A^{-1}\sigma_{e_J}^{-2}\mathbf{y}_A + \ldots + \mathbf{1}'\mathbf{D}_a^{-1}\sigma_{e_J}^{-2}\mathbf{y}_a \\ \mathbf{X}_A'\mathbf{D}_A^{-1}\sigma_{e_J}^{-2}\mathbf{y}_A + \ldots + \mathbf{X}_a'\mathbf{D}_a^{-1}\sigma_{e_J}^{-2}\mathbf{y}_a \\ \mathbf{M}_A'\mathbf{D}_A^{-1}\sigma_{e_J}^{-2}\mathbf{y}_A + \ldots + \mathbf{M}_a'\mathbf{D}_a^{-1}\sigma_{e_J}^{-2}\mathbf{y}_a \end{bmatrix} \tag{5}$$

For purebred animals,

$$\mathbf{X}_A = \begin{bmatrix} \mathbf{1} & \mathbf{0} & \mathbf{0} \end{bmatrix} \quad \mathbf{X}_B = \begin{bmatrix} \mathbf{0} & \mathbf{1} & \mathbf{0} \end{bmatrix} \quad \mathbf{X}_C = \begin{bmatrix} \mathbf{0} & \mathbf{0} & \mathbf{1} \end{bmatrix}$$

Assuming that $\mathbf{D}_A, \mathbf{D}_B, \mathbf{D}_C$ are identity matrices, and recognizing that, for example $\mathbf{1}'\mathbf{M}_A = n_A 2\mathbf{p}'_A$ and $\mathbf{M}'_A\mathbf{X}_A = \begin{bmatrix} \mathbf{M}'_A\mathbf{1} & \mathbf{0} & \mathbf{0} \end{bmatrix} = \begin{bmatrix} n_A 2\mathbf{p}_A & \mathbf{0} & \mathbf{0} \end{bmatrix}$, then equation (5) becomes:

$$\begin{bmatrix} (n_A + n_B + n_C + n_a)\,\sigma_{e_J}^{-2} & \begin{bmatrix} n_A & n_B & n_C \end{bmatrix}\sigma_{e_J}^{-2} + \mathbf{1}'\mathbf{D}_a^{-1}\mathbf{X}_a\sigma_{e_J}^{-2} & \begin{bmatrix} n_A 2\mathbf{p}'_A + n_B 2\mathbf{p}'_B + n_C 2\mathbf{p}'_C \end{bmatrix}\sigma_{e_J}^{-2} + \mathbf{1}'\mathbf{D}_a^{-1}\mathbf{M}_a\sigma_{e_J}^{-2} \\ \begin{bmatrix} n_A & n_B & n_C \end{bmatrix}'\sigma_{e_J}^{-2} + \mathbf{X}'_a\mathbf{D}_a^{-1}\mathbf{1}\sigma_{e_J}^{-2} & \begin{bmatrix} n_A & 0 & 0 \\ 0 & n_B & 0 \\ 0 & 0 & n_C \end{bmatrix}\sigma_{e_J}^{-2} + \mathbf{X}'_a\mathbf{D}_a^{-1}\mathbf{X}_a\sigma_{e_J}^{-2} & \begin{bmatrix} n_A 2\mathbf{p}_A & n_B 2\mathbf{p}_B & n_C 2\mathbf{p}_C \end{bmatrix}'\sigma_{e_J}^{-2} + \mathbf{X}'_a\mathbf{D}_a^{-1}\mathbf{M}_a\sigma_{e_J}^{-2} \\ \begin{bmatrix} n_A 2\mathbf{p}_A + n_B 2\mathbf{p}_B + n_C 2\mathbf{p}_C \end{bmatrix}\sigma_{e_J}^{-2} + \mathbf{M}'_A\mathbf{D}_A^{-1}\mathbf{1}\sigma_{e_J}^{-2} & \begin{bmatrix} n_A 2\mathbf{p}_A & n_B 2\mathbf{p}_B & n_C 2\mathbf{p}_C \end{bmatrix}\sigma_{e_J}^{-2} + \mathbf{M}'_a\mathbf{D}_a^{-1}\mathbf{X}_a\sigma_{e_J}^{-2} & (\mathbf{M}'_A\mathbf{M}_A\sigma_{e_J}^{-2} + \ldots + \mathbf{M}'_a\mathbf{D}_a^{-1}\mathbf{M}_a\sigma_{e_J}^{-2} + \mathbf{B}_J^{-1}) \end{bmatrix} \begin{bmatrix} \tilde{\mu}_J \\ \tilde{\mathbf{b}}_J \\ \tilde{\boldsymbol{\beta}}_J \end{bmatrix} \tag{6}$$

$$= \begin{bmatrix} (n_A\bar{y}_A + \ldots + n_C\bar{y}_C)\,\sigma_{e_J}^{-2} + \mathbf{1}'\mathbf{D}_a^{-1}\sigma_{e_J}^{-2}\mathbf{y}_a \\ \begin{bmatrix} n_A\bar{y}_A \\ n_B\bar{y}_B \\ n_C\bar{y}_C \end{bmatrix}\sigma_{e_J}^{-2} + \mathbf{X}'_a\mathbf{D}_a^{-1}\sigma_{e_J}^{-2}\mathbf{y}_a \\ \sum\left(\mathbf{M}'_i\sigma_{e_J}^{-2}\mathbf{y}_i\right) + \mathbf{M}'_a\mathbf{D}_a^{-1}\sigma_{e_J}^{-2}\mathbf{y}_a \end{bmatrix} \tag{7}$$

where $\mathbf{p}_i$ is a column vector of allele frequencies for breed i. Using the result in equation (3), $PEC\left(\tilde{\boldsymbol{\beta}}_i\right)^{-1} \approx \left[\mathbf{M}'_i\mathbf{M}_i\sigma_{e_i}^{-2} + \mathbf{B}_i^{-1}\right]$, and assuming $\sigma_{e_i}^2 = \sigma_{e_J}^2$, equation (6) can be re-written as:

$$\begin{bmatrix} (n_A + n_B + n_C + n_a)\,\sigma_{e_J}^{-2} & \begin{bmatrix} n_A & n_B & n_C \end{bmatrix}\sigma_{e_J}^{-2} + \mathbf{1}'\mathbf{D}_a^{-1}\mathbf{X}_a\sigma_{e_J}^{-2} & \begin{bmatrix} n_A 2\mathbf{p}'_A + n_B 2\mathbf{p}'_B + n_C 2\mathbf{p}'_C \end{bmatrix}\sigma_{e_J}^{-2} + \mathbf{1}'\mathbf{D}_a^{-1}\mathbf{M}_a\sigma_{e_J}^{-2} \\ \begin{bmatrix} n_A & n_B & n_C \end{bmatrix}'\sigma_{e_J}^{-2} + \mathbf{X}'_a\mathbf{D}_a^{-1}\mathbf{1}\sigma_{e_J}^{-2} & \begin{bmatrix} n_A & 0 & 0 \\ 0 & n_B & 0 \\ 0 & 0 & n_C \end{bmatrix}\sigma_{e_J}^{-2} + \mathbf{X}'_a\mathbf{D}_a^{-1}\mathbf{X}_a\sigma_{e_J}^{-2} & \begin{bmatrix} n_A 2\mathbf{p}_A & n_B 2\mathbf{p}_B & n_C 2\mathbf{p}_C \end{bmatrix}'\sigma_{e_J}^{-2} + \mathbf{X}'_a\mathbf{D}_a^{-1}\mathbf{M}_a\sigma_{e_J}^{-2} \\ \begin{bmatrix} n_A 2\mathbf{p}_A + n_B 2\mathbf{p}_B + n_C 2\mathbf{p}_C \end{bmatrix}\sigma_{e_J}^{-2} + \mathbf{M}'_A\mathbf{D}_A^{-1}\mathbf{1}\sigma_{e_J}^{-2} & \begin{bmatrix} n_A 2\mathbf{p}_A & n_B 2\mathbf{p}_B & n_C 2\mathbf{p}_C \end{bmatrix}\sigma_{e_J}^{-2} + \mathbf{M}'_a\mathbf{D}_a^{-1}\mathbf{X}_a\sigma_{e_J}^{-2} & \left(\sum PEC\left(\tilde{\boldsymbol{\beta}}_i\right)^{-1} + \mathbf{M}'_a\mathbf{D}_a^{-1}\mathbf{M}_a\sigma_{e_J}^{-2} - \sum\mathbf{B}_i^{-1} + \mathbf{B}_J^{-1}\right) \end{bmatrix} \begin{bmatrix} \tilde{\mu}_J \\ \tilde{\mathbf{b}}_J \\ \tilde{\boldsymbol{\beta}}_J \end{bmatrix} \tag{8}$$

$$= \begin{bmatrix} (n_A\bar{y}_A + \ldots + n_C\bar{y}_C)\,\sigma_{e_J}^{-2} + \mathbf{1}'\mathbf{D}_a^{-1}\sigma_{e_J}^{-2}\mathbf{y}_a \\ \begin{bmatrix} n_A\bar{y}_A \\ n_B\bar{y}_B \\ n_C\bar{y}_C \end{bmatrix}\sigma_{e_J}^{-2} + \mathbf{X}'_a\mathbf{D}_a^{-1}\sigma_{e_J}^{-2}\mathbf{y}_a \\ \sum PEC\left(\tilde{\boldsymbol{\beta}}_i\right)^{-1}\tilde{\boldsymbol{\beta}}_i + \mathbf{M}'_a\mathbf{D}_a^{-1}\sigma_{e_J}^{-2}\mathbf{y}_a \end{bmatrix} \tag{9}$$

When all the data from admixed animals and only the summary statistics from the pure breeds is available, these two information sources can be integrated within the Bayesian framework, such that the summary statistics are used to form prior distributions for the model parameters when analyzing admixed animals' data. In such a case equation (4)

reduces to:

$$\mathbf{y}_a \quad = \mathbf{1}\mu_J + \quad \mathbf{X}_a \;\; \mathbf{b}_J + \quad \mathbf{M}_a \;\; \boldsymbol{\beta}_J + \mathbf{e}_a$$

We only need the number of animals, mean phenotype and prediction error covariances from the pure breed analyses in order to form the priors. This leads to the following normal (N) or scale inverted chi-square $\left(\chi^{-2}\right)$ priors.

$$\mu_J \sim N\left\{\frac{1}{n_A+n_B+n_C}\left(n_A\overline{y}_A+\ldots+n_C\overline{y}_C\right), \frac{1}{n_A+n_B+n_C}\sigma^2_{e_J}\right\}$$

$$\mathbf{b}_J \sim N\left\{\begin{bmatrix} n_A & 0 & 0 \\ 0 & n_B & 0 \\ 0 & 0 & n_C \end{bmatrix}^{-1}\begin{bmatrix} n_A\overline{y}_A \\ n_B\overline{y}_B \\ n_C\overline{y}_C \end{bmatrix}, \begin{bmatrix} n_A & 0 & 0 \\ 0 & n_B & 0 \\ 0 & 0 & n_C \end{bmatrix}^{-1}\sigma^2_{e_J}\right\}$$

$$\boldsymbol{\beta}_J \sim N\left\{\left[\sum PEC\left(\tilde{\boldsymbol{\beta}}_i\right)^{-1} - \sum \mathbf{B}_i^{-1}\right]^{-1}\left[\sum PEC\left(\tilde{\boldsymbol{\beta}}_i\right)^{-1}\tilde{\boldsymbol{\beta}}_i\right], \left[\sum PEC\left(\tilde{\boldsymbol{\beta}}_i\right)^{-1} - \sum \mathbf{B}_i^{-1}\right]^{-1}\right\}$$

$$\boldsymbol{\beta}_J \sim N\left(\mathbf{0}, \mathbf{I}\sigma^2_{\beta_J}\right)$$

$$\mathbf{e}_a \sim N\left(\mathbf{0}, \mathbf{D}_a\sigma^2_{e_J}\right)$$

$$\sigma^2_{\beta_J} \sim \chi^{-2}(v_{\beta_J}, S_{\beta_J})$$

$$\sigma^2_{e_J} \sim \chi^{-2}(v_{e_J}, S_{e_J})$$

After some algebra, the full conditional distribution of $\boldsymbol{\beta}_a$ can be obtained as

$$N\left[\left(\mathbf{M}_a'\mathbf{D}_a^{-1}\mathbf{M}_a\sigma^{-2}_{e_J} + \sum PEC\left(\tilde{\boldsymbol{\beta}}_i\right)^{-1} - \sum \mathbf{B}_i^{-1} + \mathbf{B}_J^{-1}\right)^{-1}\left(\mathbf{M}_a'\mathbf{D}_a^{-1}\sigma^{-2}_{e_J}\mathbf{y}_a + \mathbf{B}^{-1}\boldsymbol{\mu}_\beta'\right), \left(\mathbf{M}_a'\mathbf{D}_a^{-1}\mathbf{M}_a\sigma^{-2}_{e_J} + \sum PEC\left(\tilde{\boldsymbol{\beta}}_i\right)^{-1} - \sum \mathbf{B}_i^{-1} + \mathbf{B}_J^{-1}\right)^{-1}\right]$$

where $\boldsymbol{\mu}_\beta' = \left[\sum PEC\left(\tilde{\boldsymbol{\beta}}_i\right)^{-1} - \sum \mathbf{B}_i^{-1}\right]^{-1}\left[\sum PEC\left(\tilde{\boldsymbol{\beta}}_i\right)^{-1}\tilde{\boldsymbol{\beta}}_i\right]$ and $\mathbf{B} = \left[\sum PEC\left(\tilde{\boldsymbol{\beta}}_i\right)^{-1} - \sum \mathbf{B}_i^{-1}\right]^{-1}$

2 A general multi-breed model

Assume the following BOA model for the data from three pure breeds ($i = A, B, C$):

$$\begin{aligned}
\mathbf{y} &= \mathbf{1}\mu + \mathbf{X}\mathbf{b} + \begin{bmatrix} \mathbf{M}_1 & \mathbf{M}_2 & \mathbf{M}_3 \end{bmatrix} \begin{bmatrix} \boldsymbol{\beta}_A \\ \boldsymbol{\beta}_B \\ \boldsymbol{\beta}_C \end{bmatrix} + \mathbf{e} \qquad (10) \\
\begin{bmatrix} \mathbf{y}_A \\ \mathbf{y}_B \\ \mathbf{y}_C \end{bmatrix} &= \mathbf{1}\mu + \begin{bmatrix} \mathbf{X}_A \\ \mathbf{X}_B \\ \mathbf{X}_C \end{bmatrix} \mathbf{b} + \begin{bmatrix} \mathbf{M}_A & \mathbf{0} & \mathbf{0} \\ \mathbf{0} & \mathbf{M}_B & \mathbf{0} \\ \mathbf{0} & \mathbf{0} & \mathbf{M}_C \end{bmatrix} \begin{bmatrix} \boldsymbol{\beta}_A \\ \boldsymbol{\beta}_B \\ \boldsymbol{\beta}_C \end{bmatrix} + \begin{bmatrix} \mathbf{e}_A \\ \mathbf{e}_B \\ \mathbf{e}_C \end{bmatrix}
\end{aligned}$$

where, $\begin{bmatrix} \mathbf{y}_A \\ \mathbf{y}_B \\ \mathbf{y}_C \end{bmatrix}$ is the vector of phenotypes, μ is the general mean, $\begin{bmatrix} \mathbf{X}_A \\ \mathbf{X}_B \\ \mathbf{X}_C \end{bmatrix}$ is the matrix of breed proportions, $\mathbf{b}$ is the vector of breed effects, $\mathbf{M}_i$ $(i = A, B, C)$ is the matrix of genotypes from the pure breed i, $\begin{bmatrix} \tilde{\boldsymbol{\beta}}_A \\ \tilde{\boldsymbol{\beta}}_B \\ \tilde{\boldsymbol{\beta}}_C \end{bmatrix}$ is a vector of breed-specific SNP effects with an assumed distributions of $\tilde{\boldsymbol{\beta}}_i \sim N(\mathbf{0}, \mathbf{B}_i)$ where $\mathbf{B}_i$ is usually a digaonal matrix of $\mathbf{I}\sigma^2_{\beta_i}$, $\begin{bmatrix} \mathbf{e}_A \\ \mathbf{e}_B \\ \mathbf{e}_C \end{bmatrix}$ is the vector of residuals with $\begin{bmatrix} \mathbf{e}_A \\ \mathbf{e}_B \\ \mathbf{e}_C \end{bmatrix} \sim N\left(\mathbf{0}, \mathbf{D}\sigma^2_e\right)$ and $\mathbf{D} = \begin{bmatrix} \mathbf{D}_A & \mathbf{0} & \mathbf{0} \\ \mathbf{0} & \mathbf{D}_B & \mathbf{0} \\ \mathbf{0} & \mathbf{0} & \mathbf{D}_C \end{bmatrix}$. We assume that SNP effects are uncorrelated, and therefore this is equivalent to separate analysis of the breeds under some assumptions.

For purebred animals,

$$\mathbf{X}_A = \begin{bmatrix} \mathbf{1} & \mathbf{0} & \mathbf{0} \end{bmatrix} \quad \mathbf{X}_B = \begin{bmatrix} \mathbf{0} & \mathbf{1} & \mathbf{0} \end{bmatrix} \quad \mathbf{X}_C = \begin{bmatrix} \mathbf{0} & \mathbf{0} & \mathbf{1} \end{bmatrix}$$

Because all entries in $\mathbf{M}_2$ and $\mathbf{M}_3$ for a purebred animal from breed A are zero, the system of equations that needs to be solved for equation (10) is as follows.

$$\begin{bmatrix}
\mathbf{1}'\mathbf{D}_A^{-1}\sigma_e^{-2}\mathbf{1} + \mathbf{1}'\mathbf{D}_B^{-1}\sigma_e^{-2}\mathbf{1} + \mathbf{1}'\mathbf{D}_C^{-1}\sigma_e^{-2}\mathbf{1} & \mathbf{1}'\mathbf{D}_A^{-1}\mathbf{X}_A\sigma_e^{-2} + \mathbf{1}'\mathbf{D}_B^{-1}\mathbf{X}_B\sigma_e^{-2} + \mathbf{1}'\mathbf{D}_C^{-1}\mathbf{X}_C\sigma_e^{-2} & \mathbf{1}'\mathbf{D}_A^{-1}\sigma_e^{-2}\mathbf{M}_A & \mathbf{1}'\mathbf{D}_B^{-1}\sigma_e^{-2}\mathbf{M}_B & \mathbf{1}'\mathbf{D}_C^{-1}\sigma_e^{-2}\mathbf{M}_C \\
\mathbf{X}_A'\mathbf{D}^{-1}\sigma_e^{-2}\mathbf{1} + \mathbf{X}_B'\mathbf{D}^{-1}\sigma_e^{-2}\mathbf{1} + \mathbf{X}_C'\mathbf{D}^{-1}\sigma_e^{-2}\mathbf{1} & \mathbf{X}_A'\mathbf{D}^{-1}\sigma_e^{-2}\mathbf{X}_A + \mathbf{X}_B'\mathbf{D}^{-1}\sigma_e^{-2}\mathbf{X}_B + \mathbf{X}_C'\mathbf{D}^{-1}\sigma_e^{-2}\mathbf{X}_C & \mathbf{X}_A'\mathbf{D}_A^{-1}\sigma_e^{-2}\mathbf{M}_A & \mathbf{X}_B'\mathbf{D}_B^{-1}\sigma_e^{-2}\mathbf{M}_B & \mathbf{X}_C'\mathbf{D}_C^{-1}\sigma_e^{-2}\mathbf{M}_C \\
\mathbf{M}_A'\mathbf{D}^{-1}\sigma_e^{-2}\mathbf{1} & \mathbf{M}_A'\mathbf{D}^{-1}\sigma_e^{-2}\mathbf{X}_A & \mathbf{M}_A'\mathbf{D}_A^{-1}\mathbf{M}_A\sigma_e^{-2} + \mathbf{B}_A^{-1} & \mathbf{0} & \mathbf{0} \\
\mathbf{M}_B'\mathbf{D}^{-1}\sigma_e^{-2}\mathbf{1} & \mathbf{M}_B'\mathbf{D}^{-1}\sigma_e^{-2}\mathbf{X}_B & \mathbf{0} & \mathbf{M}_B'\mathbf{D}_B^{-1}\mathbf{M}_B\sigma_e^{-2} + \mathbf{B}_B^{-1} & \mathbf{0} \\
\mathbf{M}_C'\mathbf{D}^{-1}\sigma_e^{-2}\mathbf{1} & \mathbf{M}_C'\mathbf{D}^{-1}\sigma_e^{-2}\mathbf{X}_C & \mathbf{0} & \mathbf{0} & \mathbf{M}_C'\mathbf{D}_C^{-1}\mathbf{M}_C\sigma_e^{-2} + \mathbf{B}_C^{-1}
\end{bmatrix}
\begin{bmatrix} \tilde{\mu} \\ \tilde{\mathbf{b}} \\ \tilde{\boldsymbol{\beta}}_A \\ \tilde{\boldsymbol{\beta}}_B \\ \tilde{\boldsymbol{\beta}}_C \end{bmatrix}$$

$$= \begin{bmatrix}
\mathbf{1}'\mathbf{D}_A^{-1}\sigma_e^{-2}\mathbf{y}_A + \mathbf{1}'\mathbf{D}_B^{-1}\sigma_e^{-2}\mathbf{y}_B + \mathbf{1}'\mathbf{D}_C^{-1}\sigma_e^{-2}\mathbf{y}_C \\
\mathbf{X}_A'\mathbf{D}_A^{-1}\sigma_e^{-2}\mathbf{y}_A + \mathbf{X}_B'\mathbf{D}_B^{-1}\sigma_e^{-2}\mathbf{y}_B + \mathbf{X}_C'\mathbf{D}_C^{-1}\sigma_e^{-2}\mathbf{y}_C \\
\mathbf{M}_A'\mathbf{D}_A^{-1}\sigma_e^{-2}\mathbf{y}_A \\
\mathbf{M}_B'\mathbf{D}_B^{-1}\sigma_e^{-2}\mathbf{y}_B \\
\mathbf{M}_C'\mathbf{D}_C^{-1}\sigma_e^{-2}\mathbf{y}_C
\end{bmatrix} \qquad (11)$$

Now let us augment data from admixed individuals (a) which leads to analysis using all available data in a BOA model:

$$\begin{aligned}
\begin{bmatrix} \mathbf{y} \\ \mathbf{y}_a \end{bmatrix} &= \mathbf{1}\mu_{BOA} + \begin{bmatrix} \mathbf{X} \\ \mathbf{X}_a \end{bmatrix} \mathbf{b}_{BOA} + \begin{bmatrix} \mathbf{M}_1 \\ \mathbf{M}_{a,1} \end{bmatrix} \boldsymbol{\beta}_{BOA,A} + \begin{bmatrix} \mathbf{M}_2 \\ \mathbf{M}_{a,2} \end{bmatrix} \boldsymbol{\beta}_{BOA,B} + \begin{bmatrix} \mathbf{M}_3 \\ \mathbf{M}_{a,3} \end{bmatrix} \boldsymbol{\beta}_{BOA,C} + \begin{bmatrix} \mathbf{e} \\ \mathbf{e}_a \end{bmatrix} \qquad (12) \\
\begin{bmatrix} \mathbf{y} \\ \mathbf{y}_a \end{bmatrix} &= \mathbf{1}\mu_{BOA} + \begin{bmatrix} \mathbf{X} \\ \mathbf{X}_a \end{bmatrix} \mathbf{b}_{BOA} + \begin{bmatrix} \mathbf{M}_1 & \mathbf{M}_2 & \mathbf{M}_3 \\ \mathbf{M}_{a,1} & \mathbf{M}_{a,2} & \mathbf{M}_{a,3} \end{bmatrix} \begin{bmatrix} \boldsymbol{\beta}_{BOA,A} \\ \boldsymbol{\beta}_{BOA,B} \\ \boldsymbol{\beta}_{BOA,C} \end{bmatrix} + \begin{bmatrix} \mathbf{e} \\ \mathbf{e}_a \end{bmatrix}
\end{aligned}$$

where $\boldsymbol{\beta}_{BOA,i}$ $(i = A, B, C)$ is the vector of breed-specific SNP effects of breed i, estimated from the analysis of full data, and we assumed that $\tilde{\boldsymbol{\beta}}_{BOA,i} \sim N\left(\mathbf{0}, \mathbf{B}_{BOA,i}\right)$ where where $\mathbf{B}_{BOA,i}$ is usually a digaonal matrix of $\mathbf{I}\sigma^2_{\beta_{BOA,i}}$ and that $\begin{bmatrix} \mathbf{e} \\ \mathbf{e}_a \end{bmatrix} \sim N\left(\mathbf{0}, \begin{bmatrix} \mathbf{D} & \mathbf{0} \\ \mathbf{0} & \mathbf{D}_a \end{bmatrix} \sigma^2_e\right)$. The following system of equations needs to be solved:

$$
\left[\begin{array}{cc}
\mathbf{1}'\mathbf{D}_A^{-1}\sigma_e^{-2}\mathbf{1} + \mathbf{1}'\mathbf{D}_B^{-1}\sigma_e^{-2}\mathbf{1} + \mathbf{1}'\mathbf{D}_C^{-1}\sigma_e^{-2}\mathbf{1} + \mathbf{1}'\mathbf{D}_a^{-1}\sigma_e^{-2}\mathbf{1} & \mathbf{1}'\mathbf{D}_A^{-1}\mathbf{X}_A\sigma_e^{-2} + \mathbf{1}'\mathbf{D}_B^{-1}\mathbf{X}_B\sigma_e^{-2} + \mathbf{1}'\mathbf{D}_C^{-1}\mathbf{X}_C\sigma_e^{-2} + \mathbf{1}'\mathbf{D}_a^{-1}\sigma_e^{-2}\mathbf{X}_a \\
\mathbf{X}_A'\mathbf{D}_A^{-1}\sigma_e^{-2}\mathbf{1} + \mathbf{X}_B'\mathbf{D}_B^{-1}\sigma_e^{-2}\mathbf{1}_B + \mathbf{X}_C'\mathbf{D}_C^{-1}\sigma_e^{-2}\mathbf{1} + \mathbf{X}_a'\mathbf{D}_a^{-1}\sigma_e^{-2}\mathbf{1} & \mathbf{X}_A'\mathbf{D}_A^{-1}\sigma_e^{-2}\mathbf{X}_A + \mathbf{X}_B'\mathbf{D}_B^{-1}\sigma_e^{-2}\mathbf{X}_B + \mathbf{X}_C'\mathbf{D}_C^{-1}\sigma_e^{-2}\mathbf{X}_C + \left(\mathbf{X}_a'\mathbf{D}_a^{-1}\sigma_e^{-2}\mathbf{X}_a\right) \\
\left(\mathbf{M}_A'\mathbf{D}_A^{-1}\sigma_e^{-2}\mathbf{1}\right) + \left(\mathbf{M}_{a,1}'\mathbf{D}_a^{-1}\sigma_e^{-2}\mathbf{1}\right) & \left(\mathbf{M}_A'\mathbf{D}_A^{-1}\sigma_e^{-2}\mathbf{X}_A\right) + \left(\mathbf{M}_{a,1}'\mathbf{D}_a^{-1}\sigma_e^{-2}\mathbf{X}_a\right) \\
\left(\mathbf{M}_B'\mathbf{D}_B^{-1}\sigma_e^{-2}\mathbf{1}\right) + \left(\mathbf{M}_{a,2}'\mathbf{D}_a^{-1}\sigma_e^{-2}\mathbf{1}\right) & \left(\mathbf{M}_B'\mathbf{D}_B^{-1}\sigma_e^{-2}\mathbf{X}_B\right) + \left(\mathbf{M}_{a,2}'\mathbf{D}_a^{-1}\sigma_e^{-2}\mathbf{X}_a\right) \\
\left(\mathbf{M}_C'\mathbf{D}_C^{-1}\sigma_e^{-2}\mathbf{1}\right) + \left(\mathbf{M}_{a,3}'\mathbf{D}_a^{-1}\sigma_e^{-2}\mathbf{1}\right) & \left(\mathbf{M}_C'\mathbf{D}_C^{-1}\sigma_e^{-2}\mathbf{X}_C\right) + \left(\mathbf{M}_{a,3}'\mathbf{D}_a^{-1}\sigma_e^{-2}\mathbf{X}_a\right)
\end{array}\right.
$$

$$
\left.\begin{array}{ccc}
\mathbf{1}'\mathbf{D}_A^{-1}\sigma_e^{-2}\mathbf{M}_A & \mathbf{1}'\mathbf{D}_B^{-1}\sigma_e^{-2}\mathbf{M}_B & \mathbf{1}'\mathbf{D}_C^{-1}\sigma_e^{-2}\mathbf{M}_C \\
\left(\mathbf{X}_A'\mathbf{D}_A^{-1}\sigma_e^{-2}\mathbf{M}_A\right) + \left(\mathbf{X}_a'\mathbf{D}_a^{-1}\sigma_e^{-2}\mathbf{M}_{a,1}\right) & \cdots & \left(\mathbf{X}_C'\mathbf{D}^{-1}\sigma_e^{-2}\mathbf{M}_C\right) + \left(\mathbf{X}_a'\mathbf{D}^{-1}\sigma_e^{-2}\mathbf{M}_{a,3}\right) \\
\left(\mathbf{M}_A'\mathbf{D}_A^{-1}\mathbf{M}_A\sigma_e^{-2} + \mathbf{B}_A^{-1}\right) + \left(\mathbf{M}_{a,1}'\mathbf{D}_a^{-1}\mathbf{M}_{a,1}\sigma_e^{-2} - \mathbf{B}_A^{-1} + \mathbf{B}_{BOA,A}^{-1}\right) & \mathbf{0} & \mathbf{0} \\
\mathbf{0} & \ddots & \mathbf{0} \\
\mathbf{0} & \mathbf{0} & \left(\mathbf{M}_C'\mathbf{D}_C^{-1}\mathbf{M}_C\sigma_e^{-2} + \mathbf{B}_C^{-1}\right) + \left(\mathbf{M}_{a,3}'\mathbf{D}_a^{-1}\mathbf{M}_{a,3}\sigma_e^{-2} - \mathbf{B}_C^{-1} + \mathbf{B}_{BOA,C}^{-1}\right)
\end{array}\right]
\begin{bmatrix} \tilde{\mu}_{BOA} \\ \tilde{\mathbf{b}}_{BOA} \\ \tilde{\boldsymbol{\beta}}_{BOA,A} \\ \tilde{\boldsymbol{\beta}}_{BOA,B} \\ \tilde{\boldsymbol{\beta}}_{BOA,C} \end{bmatrix}
$$

$$
= \begin{bmatrix}
\mathbf{1}'\mathbf{D}_A^{-1}\sigma_e^{-2}\mathbf{y}_A + \mathbf{1}'\mathbf{D}_B^{-1}\sigma_e^{-2}\mathbf{y}_B + \mathbf{1}'\mathbf{D}_C^{-1}\sigma_e^{-2}\mathbf{y}_C + \mathbf{1}_a'\mathbf{D}_a^{-1}\sigma_e^{-2}\mathbf{y}_a \\
\mathbf{X}_A'\mathbf{D}_A^{-1}\sigma_e^{-2}\mathbf{y}_A + \mathbf{X}_B'\mathbf{D}_B^{-1}\sigma_e^{-2}\mathbf{y}_B + \mathbf{X}_C'\mathbf{D}_C^{-1}\sigma_e^{-2}\mathbf{y}_C + \mathbf{X}_a'\mathbf{D}_a^{-1}\sigma_e^{-2}\mathbf{y}_a \\
\mathbf{M}_A'\mathbf{D}_A^{-1}\sigma_e^{-2}\mathbf{y}_A + \mathbf{M}_{a,1}'\sigma_e^{-2}\mathbf{y}_a \\
\mathbf{M}_B'\mathbf{D}_B^{-1}\sigma_e^{-2}\mathbf{y}_B + \mathbf{M}_{a,2}'\sigma_e^{-2}\mathbf{y}_a \\
\mathbf{M}_C'\mathbf{D}_C^{-1}\sigma_e^{-2}\mathbf{y}_C + \mathbf{M}_{a,3}'\sigma_e^{-2}\mathbf{y}_a
\end{bmatrix} \tag{13}
$$

Assuming that $\mathbf{D}_A, \mathbf{D}_B, \mathbf{D}_C$ are identity matrices, and recognizing that, for example $\mathbf{M}_A'\mathbf{X}_A = \begin{bmatrix} \mathbf{M}_A'\mathbf{1} & \mathbf{0} & \mathbf{0} \end{bmatrix} = \begin{bmatrix} n_A 2\mathbf{p}_A & \mathbf{0} & \mathbf{0} \end{bmatrix}$, then equation 13 becomes:

$$
\left[\begin{array}{cc}
\left(n_A + n_B + n_C + n_a\right)\sigma_e^{-2} & \begin{bmatrix} n_A & n_B & n_C \end{bmatrix}\sigma_e^{-2} + \mathbf{1}'\mathbf{D}_a^{-1}\mathbf{X}_a\sigma_e^{-2} \\
\begin{bmatrix} n_A & n_B & n_C \end{bmatrix}'\sigma_e^{-2} + \mathbf{X}_a'\mathbf{D}_a^{-1}\mathbf{1}\sigma_e^{-2} & \begin{bmatrix} n_A & 0 & 0 \\ 0 & n_B & 0 \\ 0 & 0 & n_C \end{bmatrix}\sigma_e^{-2} + \mathbf{X}_a'\mathbf{D}_a^{-1}\mathbf{X}_a\sigma_e^{-2} \\
n_A 2\mathbf{p}_A & \begin{bmatrix} n_A 2\mathbf{p}_A & \mathbf{0} & \mathbf{0} \end{bmatrix}\sigma_e^{-2} + \mathbf{M}_{a,1}'\mathbf{D}_a^{-1}\sigma_e^{-2}\mathbf{X}_a \\
n_B 2\mathbf{p}_B & \begin{bmatrix} \mathbf{0} & n_B 2\mathbf{p}_B & \mathbf{0} \end{bmatrix}\sigma_e^{-2} + \mathbf{M}_{a,2}'\mathbf{D}_a^{-1}\sigma_e^{-2}\mathbf{X}_a \\
n_C 2\mathbf{p}_C & \begin{bmatrix} \mathbf{0} & \mathbf{0} & n_C 2\mathbf{p}_C \end{bmatrix}\sigma_e^{-2} + \mathbf{M}_{a,3}'\mathbf{D}_a^{-1}\sigma_e^{-2}\mathbf{X}_a
\end{array}\right.
$$

$$
\left.\begin{matrix}
n_A 2\mathbf{p}'_A & n_B 2\mathbf{p}'_B & n_C 2\mathbf{p}'_C \\
\begin{bmatrix} n_A 2\mathbf{p}'_A \\ \mathbf{0} \\ \mathbf{0} \end{bmatrix} \sigma_e^{-2} + \mathbf{X}'_a \mathbf{D}_a^{-1} \sigma_e^{-2} \mathbf{M}_{a,1} & \cdots & \begin{bmatrix} \mathbf{0} \\ \mathbf{0} \\ n_C 2\mathbf{p}'_C \end{bmatrix} \sigma_e^{-2} + \mathbf{X}'_a \mathbf{D}^{-1} \sigma_e^{-2} \mathbf{M}_{a,3} \\
\left(\mathbf{M}'_A \mathbf{D}_A^{-1} \mathbf{M}_A \sigma_e^{-2} + \mathbf{B}_A^{-1}\right) + \left(\mathbf{M}'_{a,1} \mathbf{D}_a^{-1} \mathbf{M}_{a,1} \sigma_e^{-2} - \mathbf{B}_A^{-1} + \mathbf{B}_{BOA,A}^{-1}\right) & \mathbf{0} & \mathbf{0} \\
\mathbf{0} & \ddots & \mathbf{0} \\
\mathbf{0} & \mathbf{0} & \left(\mathbf{M}'_C \mathbf{D}_C^{-1} \mathbf{M}_C \sigma_e^{-2} + \mathbf{B}_C^{-1}\right) + \left(\mathbf{M}'_{a,3} \mathbf{D}_a^{-1} \mathbf{M}_{a,3} \sigma_e^{-2} - \mathbf{B}_C^{-1} + \mathbf{B}_{BOA,C}^{-1}\right)
\end{matrix}\right]
\begin{bmatrix} \tilde{\mu}_{BOA} \\ \tilde{\mathbf{b}}_{BOA} \\ \tilde{\boldsymbol{\beta}}_{BOA,A} \\ \tilde{\boldsymbol{\beta}}_{BOA,B} \\ \tilde{\boldsymbol{\beta}}_{BOA,C} \end{bmatrix}
$$

$$
= \begin{bmatrix}
\left(n_A \overline{y}_A + \ldots + n_C \overline{y}_C\right) \sigma_e^{-2} + \mathbf{1}' \mathbf{D}_a^{-1} \sigma_e^{-2} \mathbf{y}_a \\
\begin{bmatrix} n_A \overline{y}_A \\ n_B \overline{y}_B \\ n_C \overline{y}_C \end{bmatrix} \sigma_e^{-2} + \mathbf{X}'_a \mathbf{D}_a^{-1} \sigma_e^{-2} \mathbf{y}_a \\
\mathbf{M}'_A \mathbf{D}_A^{-1} \sigma_e^{-2} \mathbf{y}_A + \mathbf{M}'_{a,1} \sigma_e^{-2} \mathbf{y}_a \\
\mathbf{M}'_B \mathbf{D}_B^{-1} \sigma_e^{-2} \mathbf{y}_B + \mathbf{M}'_{a,2} \sigma_e^{-2} \mathbf{y}_a \\
\mathbf{M}'_C \mathbf{D}_C^{-1} \sigma_e^{-2} \mathbf{y}_C + \mathbf{M}'_{a,3} \sigma_e^{-2} \mathbf{y}_a
\end{bmatrix} \tag{14}
$$

where $\mathbf{p}_i$ is the column vector of allele frequencies in breed i.
Again from equation (3), it follows that

$$
\left(\mathbf{M}'_i \mathbf{M}_i \sigma_{e_i}^{-2} + \mathbf{B}_i^{-1}\right) \tilde{\boldsymbol{\beta}}_i \quad \approx \quad \mathbf{M}'_i \sigma_{e_i}^{-2} \mathbf{y}_i \tag{15}
$$

From equation (14) it follows that:

$$
\left(\mathbf{M}'_i \mathbf{D}_i^{-1} \mathbf{M}_i \sigma_e^{-2} + \mathbf{B}_i^{-1}\right) \tilde{\boldsymbol{\beta}}_{BOA,i} + \left(\mathbf{M}'_{a,j} \mathbf{D}_a^{-1} \mathbf{M}_{a,j} \sigma_e^{-2} - \mathbf{B}_i^{-1} + \mathbf{B}_{BOA,i}^{-1}\right) \tilde{\boldsymbol{\beta}}_{BOA,i} \quad = \quad \mathbf{M}'_i \mathbf{D}_i^{-1} \sigma_e^{-2} \mathbf{y}_i + \mathbf{M}'_{a,j} \mathbf{D}_a^{-1} \sigma_e^{-2} \mathbf{y}_a \tag{16}
$$

where $j = 1, 2$ or 3 for $i = A, B$ or C, respectively. Inserting (15) into (16) with the assumption that $\mathbf{D}_A, \mathbf{D}_B, \mathbf{D}_C$ and $\mathbf{D}_a$ are identity matrices, and that $\sigma_{e_i}^2 = \sigma_e^2$:

$$
\left(\mathbf{M}'_i \mathbf{M}_i \sigma_e^{-2} + \mathbf{B}_i^{-1} + \mathbf{M}'_{a,j} \mathbf{M}_{a,j} \sigma_e^{-2} - \mathbf{B}_i^{-1} + \mathbf{B}_{BOA,i}^{-1}\right) \tilde{\boldsymbol{\beta}}_{BOA,i} \quad = \quad \left(\mathbf{M}'_i \mathbf{M}_i \sigma_e^{-2} + \mathbf{B}_i^{-1}\right) \tilde{\boldsymbol{\beta}}_i + \mathbf{M}'_{a,j} \sigma_e^{-2} \mathbf{y}_a
$$

For the analysis for breed i, with $PEC\left(\tilde{\boldsymbol{\beta}}_i\right)^{-1} \approx \left[\mathbf{M}'_i \mathbf{M}_i \sigma_{e_i}^{-2} + \mathbf{B}_i^{-1}\right]$,

$$
\left[PEC\left(\tilde{\boldsymbol{\beta}}_i\right)^{-1} + \mathbf{M}'_{a,j} \mathbf{M}_{a,j} \sigma_e^{-2} - \mathbf{B}_i^{-1} + \mathbf{B}_{BOA,i}^{-1}\right] \tilde{\boldsymbol{\beta}}_{BOA,i} \quad = \quad PEC\left(\tilde{\boldsymbol{\beta}}_i\right)^{-1} \tilde{\boldsymbol{\beta}}_i + \mathbf{M}'_{a,j} \sigma_e^{-2} \mathbf{y}_a
$$

When all the data from admixed animals and only the summary statistics from the pure breeds is available, these two information sources can be integrated within the Bayesian framework, such that the summary statistics are used to form prior distributions for the model parameters when analyzing admixed animals data, as in the joint analysis. In this case, equation (12) reduces to

$$\mathbf{y}_a = \mathbf{1}\mu_{BOA} + \mathbf{X}_a\mathbf{b}_{BOA} + \begin{bmatrix} \mathbf{M}_{a,1} & \mathbf{M}_{a,2} & \mathbf{M}_{a,3} \end{bmatrix} \begin{bmatrix} \boldsymbol{\beta}_{BOA,A} \\ \boldsymbol{\beta}_{BOA,B} \\ \boldsymbol{\beta}_{BOA,C} \end{bmatrix} + \mathbf{e}_a$$

Again, we only need the number of animals, mean phenotype and prediction error covariances from the pure breed analyses. This leads to following priors for the model parameters.

$$\mu_{BOA} \sim N\left\{\frac{1}{n_A+n_B+n_C}\left(n_A\overline{y}_A+\ldots+n_C\overline{y}_C\right), \frac{1}{n_A+n_B+n_C}\sigma_e^2\right\}$$

$$\mathbf{b}_{BOA} \sim N\left\{\begin{bmatrix} n_A & 0 & 0 \\ 0 & n_B & 0 \\ 0 & 0 & n_C \end{bmatrix}^{-1}\begin{bmatrix} n_A\overline{y}_A \\ n_B\overline{y}_B \\ n_C\overline{y}_C \end{bmatrix}, \begin{bmatrix} n_A & 0 & 0 \\ 0 & n_B & 0 \\ 0 & 0 & n_C \end{bmatrix}^{-1}\sigma_e^2\right\}$$

$$\boldsymbol{\beta}_{BOA,i} \sim N\left\{\left[PEC\left(\tilde{\boldsymbol{\beta}}_i\right)^{-1} - \mathbf{B}_i^{-1}\right]^{-1}\left[PEC\left(\tilde{\boldsymbol{\beta}}_i\right)^{-1}\tilde{\boldsymbol{\beta}}_i\right], \left[PEC\left(\tilde{\boldsymbol{\beta}}_i\right)^{-1} - \mathbf{B}_i^{-1}\right]^{-1}\right\}$$

$$\boldsymbol{\beta}_{BOA,i} \sim N\left(\mathbf{0}, \mathbf{I}\sigma^2_{\beta_{BOA,i}}\right)$$

$$\mathbf{e}_a \sim N\left(\mathbf{0}, \mathbf{D}_a\sigma_e^2\right)$$

$$\sigma^2_{\beta_{BOA,i}} \sim \chi^{-2}(v_{\beta_{BOA,i}}, S_{\beta_{BOA,i}})$$

$$\sigma_e^2 \sim \chi^{-2}(v_e, S_e)$$

Full conditional distribution of $\boldsymbol{\beta}_{BOA,i}$ is

$$N\left[\left(PEC\left(\tilde{\boldsymbol{\beta}}_i\right)^{-1} + \mathbf{M}'_{a,j}\mathbf{D}_a^{-1}\mathbf{M}_{a,j}\sigma_e^{-2} - \mathbf{B}_i^{-1} + \mathbf{B}_{BOA,i}^{-1}\right)^{-1}\left(\mathbf{M}'_{a,j}\mathbf{D}_a^{-1}\sigma_e^{-2}\mathbf{y}_a + \mathbf{B}^{-1}\boldsymbol{\mu}'_\beta\right), \left(PEC\left(\tilde{\boldsymbol{\beta}}_i\right)^{-1} + \mathbf{M}'_{a,j}\mathbf{D}_a^{-1}\mathbf{M}_{a,j}\sigma_e^{-2} - \mathbf{B}_i^{-1} + \mathbf{B}_{BOA,i}^{-1}\right)^{-1}\right]$$

where $\boldsymbol{\mu}'_\beta = \left[PEC\left(\tilde{\boldsymbol{\beta}}_i\right)^{-1} - \mathbf{B}_i^{-1}\right]^{-1}\left[PEC\left(\tilde{\boldsymbol{\beta}}_i\right)^{-1}\tilde{\boldsymbol{\beta}}_i\right]$ and $\mathbf{B} = \left[PEC\left(\tilde{\boldsymbol{\beta}}_i\right)^{-1} - \mathbf{B}_i^{-1}\right]^{-1}$

References

[1] J Vandenplas, MPL Calus et G Gorjanc : Genomic prediction using individual-level data and summary statistics from multiple populations. *Genetics*, 210(1):53–69, 2018.

[2] E Karaman, G Su, I Croue et MS Lund : Genomic prediction using a reference population of multiple pure breeds and admixed individuals. *Genet Sel Evol*, 53(1):46, 2021.
